# Bacterial genome-wide association study substantiates *papGII* of *Escherichia coli* as a major risk factor for urosepsis

**DOI:** 10.1186/s13073-023-01243-x

**Published:** 2023-10-30

**Authors:** Aline Cuénod, Jessica Agnetti, Helena M. B. Seth-Smith, Tim Roloff, Denise Wälchli, Dimitri Shcherbakov, Rashid Akbergenov, Sarah Tschudin-Sutter, Stefano Bassetti, Martin Siegemund, Christian H. Nickel, Jacob Moran-Gilad, Timothy G. Keys, Valentin Pflüger, Nicholas R. Thomson, Adrian Egli

**Affiliations:** 1 Applied Microbiology Research, Department of Biomedicine, University of Basel, Basel, Switzerland; 2 Clinical Bacteriology and Mycology, University Hospital Basel, Basel, Switzerland; 3 Parasites and Microbes, Wellcome Trust Sanger Institute, Hinxton, UK; 4 Institute for Medical Microbiology, University of Zurich, Zurich, Switzerland; 5 Department of Microbiology and Immunology, McGill University, Montréal, Canada; 6 Swiss Institute for Bioinformatics, Basel, Switzerland; 7 Infectious Diseases and Hospital Epidemiology, University Hospital Basel and University of Basel, Basel, Switzerland; 8 Department of Clinical Research, University of Basel, Basel, Switzerland; 9 Division of Internal Medicine, University Hospital Basel, Basel, Switzerland; 10 Intensive Care Unit, University Hospital Basel, Basel, Switzerland; 11 Emergency Department, University Hospital Basel and University of Basel, Basel, Switzerland; 12 Department of Health Policy and Management, School of Public Health, Faculty of Health Sciences, Ben Gurion University of the Negev, Be’er Sheva, Israel; 13 Department of Health Sciences and Technology, ETH Zurich, Zurich, Switzerland; 14 Mabritec AG, Riehen, Switzerland; 15 Department of Pathogen Molecular Biology, London School of Hygiene and Tropical Medicine, London, UK

**Keywords:** *Escherichia coli*, Urinary tract infection, Invasiveness, bGWAS, *papGII*

## Abstract

**Background:**

Urinary tract infections (UTIs) are among the most common bacterial infections worldwide, often caused by uropathogenic *Escherichia coli*. Multiple bacterial virulence factors or patient characteristics have been linked separately to progressive, more invasive infections. In this study, we aim to identify pathogen- and patient-specific factors that drive the progression to urosepsis by jointly analysing bacterial and host characteristics.

**Methods:**

We analysed 1076 *E. coli* strains isolated from 825 clinical cases with UTI and/or bacteraemia by whole-genome sequencing (Illumina). Sequence types (STs) were determined via srst2 and capsule loci via fastKaptive. We compared the isolates from urine and blood to confirm clonality. Furthermore, we performed a bacterial genome-wide association study (bGWAS) (pyseer) using bacteraemia as the primary clinical outcome. Clinical data were collected by an electronic patient chart review. We concurrently analysed the association of the most significant bGWAS hit and important patient characteristics with the clinical endpoint bacteraemia using a generalised linear model (GLM). Finally, we designed qPCR primers and probes to detect *papGII*-positive *E. coli* strains and prospectively screened *E. coli* from urine samples (*n* = 1657) at two healthcare centres.

**Results:**

Our patient cohort had a median age of 75.3 years (range: 18.00–103.1) and was predominantly female (574/825, 69.6%). The bacterial phylogroups B2 (60.6%; 500/825) and D (16.6%; 137/825), which are associated with extraintestinal infections, represent the majority of the strains in our collection, many of which encode a polysaccharide capsule (63.4%; 525/825). The most frequently observed STs were ST131 (12.7%; 105/825), ST69 (11.0%; 91/825), and ST73 (10.2%; 84/825). Of interest, in 12.3% (13/106) of cases, the *E. coli* pairs in urine and blood were only distantly related. In line with previous bGWAS studies, we identified the gene *papGII* (*p*-value < 0.001), which encodes the adhesin subunit of the *E. coli* P-pilus, to be associated with ‘bacteraemia’ in our bGWAS. In our GLM, correcting for patient characteristics, *papGII* remained highly significant (odds ratio = 5.27, 95% confidence interval = [3.48, 7.97], *p*-value < 0.001). An independent cohort of cases which we screened for *papGII*-carrying *E. coli* at two healthcare centres further confirmed the increased relative frequency of *papGII*-positive strains causing invasive infection, compared to *papGII*-negative strains (*p*-value = 0.033, chi-squared test).

**Conclusions:**

This study builds on previous work linking *papGII* with invasive infection by showing that it is a major risk factor for progression from UTI to bacteraemia that has diagnostic potential.

**Supplementary Information:**

The online version contains supplementary material available at 10.1186/s13073-023-01243-x.

## Background

Urinary tract infections (UTIs) are among the most common diseases, affecting > 150 million people each year worldwide [1]. Up to 60% of women suffer from at least one symptomatic UTI in their lifetime, with ~ 10% of women experiencing a symptomatic UTI every year [2]. *Escherichia coli* is the most common cause of UTI [3]. While most UTIs cause only mild symptoms, some ascend the urinary tract and progress to cause invasive infections such as pyelonephritis, urosepsis, and septic shock [4, 5]. Invasive infections are associated with high morbidity and healthcare costs and can lead to septic shock and mortality [6, 7]. Therefore, understanding the factors associated with a more severe disease outcome is critical for patient management, to decrease healthcare-related costs and improve antibiotic stewardship.

Within *E. coli*, multiple distinct and deep-branching phylogenetic groups (defined as phylogroups) have been identified [8]. Of these A, B1, B2, and D are most common [8, 9]. Phylogroups A and B1 are associated with asymptomatic carriage in the gut while phylogroups B2, D, and F predominantly cause extraintestinal infections [10, 11] Furthermore, there are large variations in clinical phenotypes within each phylogroup. This is linked to the presence or absence of virulence and antimicrobial resistance (AMR) genes, which can differ even between closely related strains [9]. Consequently, UTIs can be caused by *E. coli* strains from multiple different phylogroups [12]. Well-characterised uropathogenic *E. coli* (UPEC) virulence factors include iron uptake systems, capsular polysaccharides, immune modulators, fimbriae, and pili [13–15]. Genes encoding these clinically important bacterial factors are associated with globally successful UPEC clones such as sequence types (ST) 131, ST69, ST73, and ST95 [5, 13, 16].

Among the known virulence factors, PapGII has an important role in the progression of UTI to invasive infection [13] and, from the perspective of invasive infection, is associated with the urinary and intestinal tracts as ports of entry for *E. coli* bacteraemia [17]. The *papGII* gene encodes one of five variants (*papGI*–*papGV*) of the adhesive tip of pyelonephritis-associated pili (PAP) [18]. Another bacterial factor which has been associated with invasive UTI is encoded by *iuc*, which is essential for the biosynthesis of the iron uptake system aerobactin [13].

The clinical course of a UTI and subsequent pyelonephritis and urosepsis is not only shaped by these bacterial factors, but also by human characteristics such as an effective immune response, host genetics, comorbidities, age, and sex [2]. As such, the clinical importance of an individual bacterial factor cannot be definitively assessed without correcting for relevant patient characteristics. Despite our growing understanding of UPEC virulence and patient risk factors, these two interconnected aspects are mostly analysed separately, and their interaction is rarely considered [17].

In this study, we aim to identify pathogen- and patient-specific factors associated with bacteraemia by jointly analysing genomic bacterial data and host characteristics from patients with *E. coli*-positive urine or blood culture samples. We further aim to investigate whether virulent UPEC isolates can accurately be identified in clinical routine diagnostics and to validate our findings in a second, prospectively collected cohort.

## Methods

### Patient data collection

To assess which patient characteristics impact the progression of a UTI, we reviewed 825 clinical case charts. Demographic and clinical data were systematically collected for patients with an *E. coli* isolated from the blood or urine between 04/2018 and 02/2020 at the University Hospital Basel by retrospective electronic chart review. The University Hospital Basel is a tertiary healthcare centre with more than 750 beds in a low endemic region for extended-spectrum β-lactamase (ESBL)-producing bacteria [19]. The inclusion criteria were patients for which at least one isolated bacterial colony was collected from urine or blood culture samples, identified as *E. coli* by MALDI-TOF MS (Bruker Daltonics, Bremen, Germany) or using biochemical assays on the Vitek2 (bioMérieux, Marcy-l’Étoile, France), and no negative statement for the hospital’s general research consent as approved by the ethical committee. The following clinical data was recorded: the presence of typical urinary tract symptoms (dysuria, increased urinary frequency, urgency [20]), mention of *E. coli* infection as a medical diagnosis, and laboratory results (including leukocytes, C-reactive protein, and urine flow cytometry) on the day (± 1) of urine and/or blood sample collection, as well as the requirement for antibiotic treatment. Information on the need for and duration of hospitalisation, admission to the ICU, and in-hospital mortality were extracted. Additionally, we determined 30-day all-cause mortality, defined as a death occurring for any reason within 30 days of the collection of the urine or blood culture sample. Relevant comorbidities were recorded according to the Charlson Comorbidity Index (CCI) [21]. Immunosuppression is defined as corticosteroid dose equivalent to prednisone 20 mg daily, active haematological malignancy history of haematopoietic stem cell transplant or solid organ transplant, or absolute neutrophil count < 500 cells/μl. We summarised patient demographic and clinical data into units of clinical cases, which were defined as a unique hospital stay and included data collected from the same patient between the hospital entry and the hospital exit date.

### Definition of ‘invasiveness’

*E. coli* strains were defined as ‘invasive’ if isolated from a blood culture or if a blood culture from the same clinical case was diagnosed positive for *E. coli* within 7 days before or after the collection of the *E. coli*-positive urine sample. Similarly, the term ‘invasive infection’ was used to describe clinical cases with at least one *E. coli*-positive blood culture sample, whereas ‘non-invasive’ cases described all other cases in our cohort. In non-invasive cases, a median of 0 blood draws was taken (interquartile range (IQR) = [0,0]), compared to invasive cases where a median of 1 blood draw was taken (IQR = [1,2]). Typically, each blood draw consists of two blood culture flasks being cultured, one aerobic and one anaerobic. In ‘invasive’ cases, a median of 2 (interquartile range (IQR) = [2,4]) blood culture samples were reported *E. coli* positive in routine diagnostics.

### Bacterial isolate collection and whole-genome sequencing

*E. coli* isolates (*n* = 1076) were prospectively collected at the University Hospital Basel from 825 clinical cases from urine (*n* = 789), blood culture (*n* = 286), and deep tissue samples (*n* = 1) from 04/2018 to 02/2020, aiming for a balanced dataset between invasive and non-invasive infections. Urine was cultured on 5% Columbia sheep blood (COS) agar plates (Becton Dickinson, NJ, USA) and Chrom ID plate (Becton Dickinson, NJ, USA) for a maximum of 48 h. Blood cultures were incubated in aerobic and anaerobic flasks for a maximum of 6 days using the Virtuo system (bioMérieux, Marcy-l’Étoile, France). Bacteria which grew in clinically relevant numbers were identified as *E. coli* in routine diagnostics using the microflex Biotyper MALDI-TOF MS system (Bruker Daltonics, Bremen, Germany). ‘Clinically relevant numbers’ were defined as ≥ 10^5^ colony-forming units/ml for urine samples (or lower when specifically ordered by the clinical personnel), whereas all positive blood cultures were regarded as clinically relevant. For whole-genome sequencing, isolates were grown on COS agar (bioMérieux, Marcy-l’Étoile, France), and DNA was extracted using the QIACube with the QIAamp DNA Mini Kit (QIAGEN, Hilden, Germany). After quality control of the DNA by Tapestation (Agilent, Santa Clara, USA), tagmentation libraries were generated as described by the manufacturer (Illumina DNA Prep Kit, Illumina, San Diego, USA). The genomes were sequenced using a 2 × 300 base pairs V3 reaction kit on an Illumina MiSeq or using a 2 × 150 base pairs on an Illumina NextSeq500 instrument. Raw reads of all isolates are publicly available via the European Nucleotide Archive (Project Accession PRJEB55855).

### Phenotypic antimicrobial susceptibility testing

Antimicrobial susceptibility testing (AST) was performed in routine diagnostics using the Vitek2 system (AST-N242 GN Cards, bioMérieux, Marcy-l’Étoile, France) or using gradient diffusion strips (Liofilchem, Roseto degli Abruzzi, Italy), and the measurements were interpreted according to EUCAST clinical breakpoints (v.9.0) into the categories ‘susceptible’, ‘intermediate’, or ‘resistant’.

### Comparative genomic analysis

We trimmed the raw reads using Trimmomatic (v0.38) [22], generated assemblies using Spades (v3.6.2) [23] via unicycler (v0.3.0) [24], and polished them using pilon (v1.23) [25]. We examined the following features to ensure the quality of the sequence data and assembly: average read quality (median = 98.4; range = [56.4, 99.5]) and depth (mean = 74.6 × ; range = [12.9 × , 328.8 ×]), %G + C content (median = 50.6; range = [50.3–51.1]), genome size (median = 5.0 megabases (MB); range = [4.4–5.9]). The purity of the sample was assessed using MetaPhlan [26], and genomes were annotated using prokka (v1.13) [27]. Bacterial species identification was confirmed using ribosomal multi-locus sequence typing (rMLST) [28], where three strains were identified as *Escherichia marmotae* and one as *Escherichia ruysiae*. These were excluded from further analysis. We screened all assemblies for the occurrence of previously described UPEC virulence (EcVGDB [13]) and resistance factors (NCBI [29]) using abricate (v0.8.10) (https://github.com/tseemann/abricate) and > 95% coverage and > 95% identity thresholds. On the basis of genomic Mash distances, the *E. coli* phylogroups have recently been suggested to be split up, resulting in 14 phylogroups, including two which correspond to strains of the genus *Shigella* [11]. In order to assign our genomes to these phylogroups, we calculated the mash (v2.2) [30] distance to the medoid reference genomes of each phylogroup [11], except for phylogroup C, where an alternative reference genome was used (GCF_001515725.1), as the published medoid reference genome clustered within phylogroup B1. We chose an alternative reference genome for phylogroup C from the Microreact project (https://microreact.org/project/10667ecoli/c38356ec) belonging to phylogroup C according to ‘PCR Phylogroup’ and ‘Mash-Screen-Phylogroup’ and having the highest ‘total score’ and ‘sequence score’. We assigned the phylogroup of the closest reference genome to each queried genome using a cut-off of 0.04 mash distance [11]. We identified the *E. coli kps* loci and capsule types using fastKaptive (v0.2.2) [31], considering the best hit per genome if a minimal coverage of 80% to a reference loci was detected and no reference gene was missing. To link the fastKaptive assignment to phenotypically assigned capsule types, we ran reference genomes for the well-known capsule types K1 (CP003034.1) and K5 (CP022686.1) through fastKaptive, which were assigned as ‘KX03’ and ‘KX29’, respectively. We determined the O- and H antigens as well as the multi-locus sequence type (MLST) via srst2 (v0.2.0) [32, 33]. We assessed the average nucleotide identity (ANI) between sequenced isolates using fastANI (v1.32) [34]. We used Panaroo (‘sensitive’ mode) (v1.2.7) [35] to identify the core genome, which we aligned using mafft (v7.467) [36] and used RaxML (GTRCAT approximation) (v8.2.8) [37] to construct a phylogenetic tree from this alignment. The output from panaroo includes a nucleotide alignment for each gene. The alignment of the gene *hdeA* was translated to amino acid sequences using transseq [38]. The signal peptide [39] was removed, before predicting the mass of the HdeA protein using protparam [40]. To investigate the diversity of *E. coli* isolates within a sample, we sequenced ten colonies picked from three blood culture samples and three urine samples each. For pairwise comparison of isolates recovered from the same patient sample, we used the variant caller Freebayes (v1.2.0) [41] via snippy (minimum mapping quality = 60, minimum base quality = 30, minimum coverage = 30, minimum proportion for variant evidence = 0.95, v4.3.6) (https://github.com/tseemann/snippy).

### Bacterial genome-wide association study

To identify the bacterial factors associated with invasive infection, we used pyseer (v1.3.9) [42]. We used unitigs as inputs and invasive infection (i.e. bacteraemia) as a clinical endpoint. Unitigs represent non-redundant sequence elements of variable lengths, which we had previously constructed via unitig-counter (v1.0.5) [43]. A minor allele frequency threshold of 5% was used. We chose not to consider rare alleles which occur in less than 5%, as we aimed to identify alleles with the biggest diagnostic potential. We treated each clinical case (*n* = 825) as a single event and included one *E. coli* genome per case in the analysis. If multiple strains per clinical case had been isolated, we chose isolates recovered from blood culture samples over those recovered from urine samples, as these caused the invasive infection. If there were multiple strains isolated from the same material, we included the isolates recovered at the earliest time point. We used random effects to correct for population structure (‘-lmm’ mode) by providing a similarity-matrix acquired from the core genome phylogeny (constructed as described above). Unitigs were mapped against the genome annotations of all strains (*n* = 825). In order to identify the *papG* variant against which the unitigs mapped, we compared these to the reference sequences for *papGI*, *papGII*, *papGIII*, *papGIV*, and *papGV* [13] using fastANI (v1.32) [34].

To focus on the urinary tract as a port of entry for invasive infection, the analysis was repeated excluding invasive strains for which no matching urine isolate was identified in our dataset (168 strains excluded, 657 strains remaining including 93 causing invasive infection and 564 not causing invasive infection).

To optimally control for confounding factors, we repeated both bacteria genome wide association studies (bGWAS) analyses using important host characteristics as well as phenotypic resistance to ceftriaxone of the infecting *E. coli* strain as covariates. These were the same host characteristics as used in the generalised linear models (GLM); please refer to the respective section for more details.

### Generalised linear models

We evaluated the impact of *papGII* on clinical outcomes by building GLMs, correcting for important patient characteristics. Such analyses are important to assess the potential of *papGII* as a diagnostic marker for virulent infection. The clinical outcomes analysed in this study included (i) invasive infection (defined as at least one *E. coli*-positive blood culture sample, see above), (ii) experience of typical UTI symptoms, (iii) admission to an intensive care unit (ICU), and (iv) all-cause mortality within 30 days of *E. coli* sample collection. Clinical outcomes were examined for an association with *papGII* carriage and phenotypic resistance to ceftriaxone of the infecting *E. coli* strain, age, sex, immunosuppression (see definition in the ‘Patient data collection’ section), and CCI [21]. We corrected for resistance to ceftriaxone, as we aimed to investigate bacterial virulence and not AMR impacting patient outcome. If multiple strains per clinical case had been isolated, we chose *papGII* carriage and ceftriaxone resistance of strains isolated from blood culture samples, over strains isolated from urine samples. If multiple strains had been isolated from the same material, we chose the *papGII* carriage and ceftriaxone resistance of the strain isolated at the earliest time point. To compare the effect size between the included variables, we scaled and centred the numerical variables ‘age’ and ‘CCI’. All outcomes were binary and were analysed using GLM with binomial error distribution. Statistical analyses were performed in R (v 3.7). We built simple GLM classifiers with ‘invasive infection’ as an outcome and used the same dataset and variables as in our GLM as input (*n* = 751 complete observations, 210 events). We built two classifiers (a) including *papGII* as a predictor and (b) omitting *papGII* as a predictor. We used fivefold cross-validation and repeated each analysis ten times. The mean and standard deviations (sd) of the following readout variables were assessed: accuracy, area under the receiver operating curve (AUROC), sensitivity, specificity, positive predictive value, and negative predictive value. Analyses were performed in R (v 3.7) and using the package ‘caret’ [44].

### MALDI-TOF MS

We aimed to identify virulent *E. coli* strains using MALDI-TOF MS. We acquired MALDI-TOF mass spectra for a subset of *E. coli* isolates (*n* = 303). This subset represents strains encoding *papGII* (*n* = 78) and strains which did not encode *papGII* (*n* = 225), as well as representative isolates of the phylogroups A (*n* = 19), B1 (*n* = 26), B2-1 (*n* = 48), B2-2 (*n* = 135), C (*n* = 7), D1 (*n* = 42), D2 (*n* = 5), D3 (*n* = 13), and F (*n* = 8). Each strain was measured in quadruplicate on two MALDI-TOF MS devices including a Microflex Biotyper ‘smart’ (Bruker Daltonics, Bremen, Germany) and an Axima Confidence (Shimadzu, Ngoyo, Japan) using direct smear method and overlaying with 1μl formic acid (25%) and 1μl cyano-4-hydroxycinnamic acid (CHCA) matrix solution.

Mass spectra acquired on the Axima Confidence were exported as ‘mzXml’ and mass spectra acquired on the microflex Biotyper as ‘fid’ files, and both were further processed in R using the packages MALDIQuant and MALDIQuantForeign [45]: mass spectra were trimmed to a mass range of 4000–20,000, the intensity was transformed (‘sqrt’) and smoothed (method = “SavitzkyGolay”, halfWindowSize = 20), the baseline was removed (method = “SNIP”, 40 and 160 iterations for spectra acquired on the microflex Biotyper or the Axima Confidence, respectively), and the intensity was calibrated (method = “median”) before peaks were detected (“SuperSmoother”, halfWindowSize = 20, SNR = 2). Peaks were calibrated by aligning the mass spectra to 23 conserved masses (4364.4 Da, 5095.8 Da, 6371.5 Da, 6446.3 Da, 6541.7 Da, 7273.4 Da, 7288.8 Da, 8499.9 Da, 9006.4 Da, 9704.3 Da, 10,430.2 Da, 11,564.2 Da, 11,580.4 Da, 11,735.4 Da, 12,769.5 Da, 13,133.1 Da, 13,540.9 Da, 14,126.4 Da, 14,875.2 Da, 15,281.0 Da, 15,768.9 Da, 17,603.2 Da, 17,711.4 Da) and 1000 ppm tolerance in both directions. All scripts can be accessed via GitHub (https://github.com/acuenod111/UPEC) [46]. All raw mass spectra and processed peak lists can be accessed via the Open Science Foundation (https://osf.io/vmqc5/) [47].

### Real-time polymerase chain reaction (qPCR)

To substantiate our findings, we prospectively collected a second, independent set of urine and blood culture samples from two different healthcare centres: the University Hospital Basel between 05/2022 and 06/2022 (= centre 1) and the Institute of Medical Microbiology of the University of Zurich between 09/2022 and 02/2023 (= centre 2).

We screened these samples for the presence of *papGII* carrying *E. coli* using qPCR. Our assay included primers (i) for *E. coli* specific core genes (*gapDH-C* [48–50] and *uidA* [51] at centre 1 and *rpoD* at centre 2); (ii) for *papC*, to detect the *pap*-operon; and (iii) for *papGII* to specifically detect this variant of *papG*. The sequences of the primers and probes can be found in Additional file 1: Supplementary Methods. We verified whether there were variants of these sequences in our set of genomes (*n* = 1076) using the variant caller Freebayes (v1.2.0) [41] via snippy (minimum mapping quality = 60, minimum base quality = 30, minimum coverage = 30, minimum proportion for variant evidence = 0.95, v4.3.6) (https://github.com/tseemann/snippy). We further tested the performance of our assay when being applied directly to urine sample pellets, thereby decreasing turnaround time by omitting cultivation. We assessed the efficiency and the limit of detection of our qPCR assay performed directly on samples and compared them to values resulting from qPCR on extracted genomic DNA.

We used the established qPCR method to prospectively screen clinical urine samples (*n* = 543) at centre 1. This included urine samples collected in routine diagnostics, including 261 samples of 244 cases, which were culture-positive for *E. coli*. In contrast at centre 2, we did not perform our qPCR directly from urine samples, but from *E. coli* cultures isolated from urine or from blood culture samples in routine diagnostics (1128 samples from 886 clinical cases). We evaluated which clinical cases progressed to an invasive infection, which we defined as a positive blood culture within 7 days after the retrieval of the urine sample. More details can be found in Additional file 1: Supplementary Methods.

## Results

### Patient factors

**Fig. 1:**
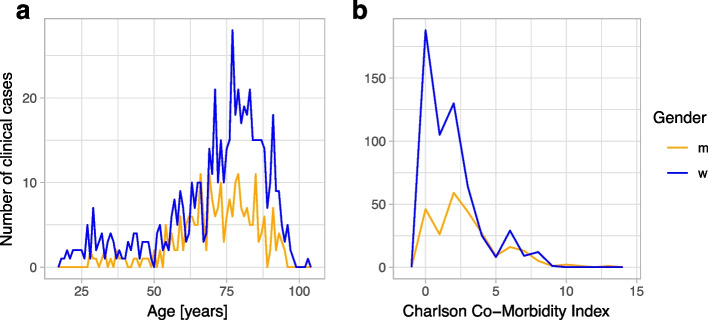
Frequency distribution of **a** age [years] and **b** the sum of the Charlson Comorbidity Index (CCI) for male (orange) and female (blue) clinical cases (*n* = 825) included in this study

To identify the most relevant patient characteristics for the progression of a UTI, we reviewed 825 clinical case charts. This included cases for which *E. coli* isolates were identified from urine (*n* = 564) or from blood culture samples (*n* = 261). In 106 cases, *E. coli* isolates were recovered from urine and blood culture samples, suggesting the urinary tract as a port of entry for bacteraemia. Cases for which *E. coli* isolates were recovered from blood culture samples are henceforward referred to as ‘invasive infection’, compared to ‘non-invasive infection’ where *E. coli* isolates were recovered from urine samples, but not from blood culture samples. Patients had a median age of 75.3 years (IQR = [63.6, 83.0]) and a median CCI of 2 (IQR = [0, 3]) and were predominantly female (69.6%, 574/825), and 10.6% (86/812) were immunosuppressed (see definition in the ‘Patient data collection’ section) (Fig. 1, Table 1).

**Table 1:** Patient characteristics and outcome variables of clinical cases with 261/825 invasive or 564/825 non-invasive infections. ‘Invasive infection’ was defined as at least one positive *E. coli* blood culture, whereas ‘non-invasive infection’ was defined as at least one *E. coli*-positive urine sample, with no blood culture being tested positive for *E. coli*. Some variables were unavailable for a subset of patients, resulting in varying denominators in the table. The numeric values ‘age’ and ‘CCI’ were compared using a Mann–Whitney *U* test and the factorial variables ‘sex’, ‘immunosuppression’, ‘typical UTI symptoms, ‘ICU admission’, and ‘30-day all-cause mortality’ using a Chi-squared test

Variable	All cases (*n* = 825)	Cases with invasive infection (*n* = 261)	Cases with non-invasive infection (*n* = 564)	*p*-value invasive vs. non-invasive cases
Age [years] (median, [IQR])	75.3 [63.6,83.0]	74.7 [63.7, 82.3]	75.7 [63.4, 83.3]	5.5 × 10^−1^ (ns)
Sex = male (*n* (%))	251/825 (30.4)	120/261 (46.0)	131/564 (23.2)	6.83 × 10^−11^ (***)
CCI (median [IQR])	2 [0, 3]	2 [0, 3]	2 [0, 3]	9.3 × 10^−1^ (ns)
Immunosuppression = *true* (*n* (%))	86/811 (10.6)	44/256 (17.2)	42/555 (7.6)	6.00 × 10^−5^ (***)
Typical UTI symptoms	231/778 (29.7)	77/256 (30.1)	154/522 (29.5)	9.3 × 10^−1^ (ns)
ICU admission	188/821 (22.9)	63/260 (24.2)	125/561 (22.23)	6.0 × 10^−1^ (ns)
30-day all-cause mortality	50/819 (6.1)	21/258 (8.1)	29/561 (5.2)	1.4 × 10^−1^ (ns)

*ns* not significant, *CCI* Charlson Comorbidity Index, *UTI* urinary tract infection, *ICU* intensive care unit

^***^p-value < 0.001

We observed a larger fraction of rare STs (302/574, 52.6% vs. 100/251, 39.8%, *p*-value = 0.0010, Chi-squared test) and a similar fraction of isolates belonging to carriage-associated phylogroups A, B1, and C (120/574, 20.9% vs. 42/251, 16.7% *p*-value = 0.196, Chi-squared test) isolated from female patients compared to male patients, whereas male patients more frequently carried strains of ST131 than female patients (49/251, 19.5% vs. 56/574, 9.8%, *p*-value = 0.0002, Chi-squared test) (Additional file 1: Fig. S1). We observed a non-significant tendency of female patients younger than 40 years (*n* = 52) being less frequently infected with isolates from carriage-associated phylogroups than female patients older than 40 years (6/52, 11.5% vs. 114/522, 21.8%, *p*-value = 0.118, Chi-squared test). The most frequent STs isolated from female patients younger than 40 were from ST69 (8/52, 15.4%) and ST95 (7/52, 13.5%), both of which occurred in lower frequency in female patients older than 40 (55/522, 10.5% and 22/522, 4.2%, respectively) (Additional file 1: Fig. S2).

The composition of phylogroups isolated from invasive and non-invasive infection was similar with 19.2% (50/261) and 19.9% (112/564) (0.200 Chi-squared test) being caused by carriage-associated phylogroups A, B1, and C. The three most common STs were the same in both invasive and non-invasive infection, namely ST69 (38/261, 14.6% and 53/564, 9.4%), ST73 (33/261, 12.6% and 51/564, 9.0%), and ST131 (34/261, 13.0% and 71/564, 12.6%). Rare STs, which overall occurred less than 20 times in our strain collection, together caused a smaller fraction of invasive infections than non-invasive infections (109/261, 41.8% vs. 293/564 52.0%, *p*-value = 00.0081, Chi-squared test) (Additional file 1: Fig. S3).

Overall cases where this information was available from the patient records, in 29.7% (231/778) of cases, the patient experienced typical UTI symptoms, 22.9% (188/821) were admitted to the ICU, and 6.1% (50/819) died within 30 days after the collection of the urine or blood culture sample (Table 1).

### Characterisation of bacterial strains

**Fig. 2:**
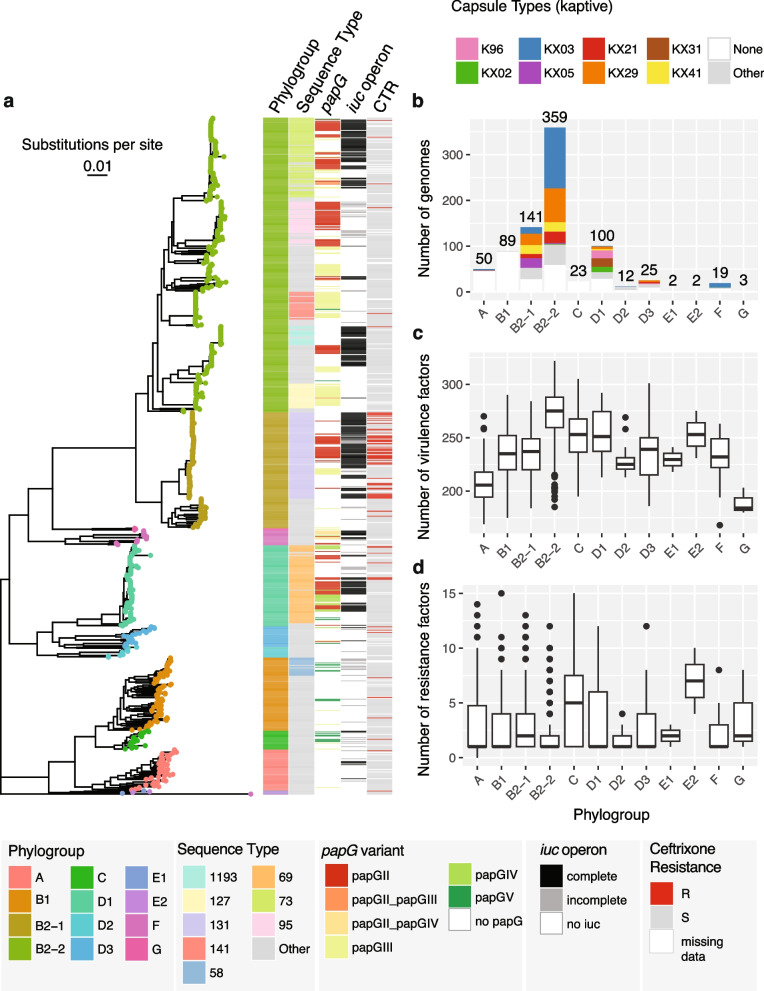
Genomic characterisation of the *E. coli* strains collected for this study (one strain per clinical case, *n* = 825). **a** Core genome phylogeny, phylogroup assignment, sequence type (ST) (eight most frequent ST are coloured, rare STs in grey), *papG* variant, occurrence of the *iuc* operon, and phenotypic ceftriaxone resistance. **b** Frequency distribution of the *E. coli* phylogroups and their respective capsule types. **c** Number of virulence factors detected per *E. coli* phylogroup. **d** Number of genes associated with antimicrobial resistance per *E. coli* phylogroup

STs which comprise globally successful UPEC clones [5, 13, 16, 52] were frequent in our collection of isolates, namely ST131 (12.7%; 105/825), ST69 (11.0%; 91/825), ST73 (10.2%; 84/825), and ST95 (5.2%; 43/825) being the four most prevalent STs (Fig. 2a). We further screened our isolates for the presence of *papGII* and the *iuc* operon, which have previously been identified as important factors in the progression of UTI [13, 17]. We detected *papGII* in 20.7% of isolates (171/825) and exclusively in the ExPEC-associated phylogroups B2-1 (20.6%; 29/141), B2-2 (27.0%; 97/359), D1 (32.0%; 32/100), D3 (8.0%; 2/25), and F (57.9%; 11/19). *papGII* frequently occurs within closely related strains [13] (Fig. 2a). The *iuc* operon often co-occurs with *papGII* and less frequently with other *papG* variants. The complete *iuc* operon was detected in 28.8% (238/825) of all strains and in the phylogroups A (10%; 5/50), B2-1 (52.5%; 74/141), B2-2 (32.0%; 115/359), C (8.7%; 2/23), D1 (30.0%; 31/100), D3 (8%; 2/25), and F (47.4%; 9/19).

Phenotypic ceftriaxone resistance, which is often assessed to screen for the carriage of ESBL, was rare in our dataset (9.8%; 72/737), except in isolates of ST131 (51.6%, 48/93) (Fig. 2a). The most frequently detected ESBL genes detected in the ceftriaxone-resistant strains were *blaCTX-M-15* (54.2%; 39/72) and *blaCTX-M-14* (8.3%; 6/72). In 23.6% (17/72) of these resistant strains, no ESBL gene was detected. An ESBL gene was however detected in 176 strains which tested sensitive to ceftriaxone. The most frequent of these were the serine beta lactamases *blaEC-18* (69/176), *blaEC-19* (60/176), *blaEC-13* (28/176), and *blaEC-15* (16/176).

We did not record any phenotypic resistance against meropenem (0/737), and phenotypic resistance against fosfomycin and nitrofurantoin was rare (1.5%; 8/547 and 1.1%; 6/546) and distributed throughout the phylogenetic tree (Additional file 1: Fig. S4). Phenotypic resistance against ciprofloxacin was more prevalent (17.0%; 125/737) and occurred most often in ST1193 (100%; 18/18) and ST131 isolates (67.7%; 63/93) (Additional file 1: Fig. S4).

We observed a large fraction of our isolates (79.5%; 656/825) belonging to the ExPEC-associated phylogroups B2-2 (43.5%; 359/825), B2-1 (17.1%; 141/825), D1 (12.1%; 100/825), D2 (1.5%; 12/825), D3 (3.0%; 25/825), and F (2.3%; 19/825). Only a smaller fraction of strains belonged to phylogroups associated with colonisation: A (6.1%; 50/825), B1 (10.8%; 89/825), C (2.8%; 23/825), and/or intestinal infection: E2 (0.2%; 2/825) (Fig. 2a). We observed 63.4% (525/825) of the strains carrying genes encoding capsular polysaccharides. Strains in ExPEC-associated phylogroups most often encoded a capsule (79.1%; 519/656) whereas strains in phylogroups associated with colonisation rarely did (3.7%; 6/162). The most common capsule assignments were ‘KX03’ (30.7%; 161/525) and ‘KX29’ (20.6%; 108/525). These are the same capsule assignments, which are assigned to strains expressing the well-known capsule types K1 and K5 (Fig. 2b).

We next compared the phylogroups in terms of virulence factors and resistance genes. Of the phylogroups which included more than 10 isolates, phylogroup A strains carried the fewest virulence factors (median = 205.5, IQR = [194.25; 217.75]), while phylogroup B2-2 strains carried the most virulence factors (median = 275.0; IQR = [259.0, 288.0]) (Fig. 2c).

Phylogroups A, B1, B2-2, D1, D2, D3, and F carried a median of one resistance gene. Of the phylogroups which cover more than 10 isolates, phylogroup C harboured the most resistance genes (median = 5; IQR = [1, 7.5]), followed by B2-1 (median = 2; IQR = [1, 4]) (Fig. 2d).

### Genetic diversity of *E. coli* isolated from the same human host

Whereas it would be expected to find almost identical isolates in cases where *E. coli* strains were isolated from urine and blood cultures of the same patient, we found two phylogenetically distant strains with average nucleotide identity (ANI) values under 99.9% in 12.3% (13/106) of cases (Additional file 1: Fig. S5a). In 11/13 of these cases, these distantly related strains encoded varying *papG* variants with the blood culture isolates more frequently encoding *papGII* than the urine isolates of the same cases (5/13 vs. 0/13) (Additional file 1: Fig. S5b). To compare the strain diversity within urine and blood culture samples, we picked ten colonies for single-colony sequencing each from urine and blood culture samples of three patients with *E. coli* bacteraemia. In one of the three cases, we observed two distinct subpopulations in urine, where 6/10 isolates cluster apart from the remaining 4/10 urine isolates and the 10/10 blood culture isolates. None of these strains encodes *papG*. Strains between these distinct subpopulations share 96.8–96.9% ANI and display 71,193– 80,916 single nucleotide variants (SNV) in pairwise comparisons (Additional file 1: Fig. S5b, Fig. S5c). When pairwise examining closely related strains from the same sample (over 99.9 ANI), we identified a higher number of SNVs (median = 3, IQR = [2, 5]) for strains co-isolated from urine samples than for strains co-isolated from blood culture samples (median = 2, IQR = [0, 2], *p*-value = 0.00033, Mann–Whitney *U* test (Additional file 1: Fig. S5b).

### Bacterial genome-wide association study

To identify bacterial factors promoting UTI progression, we performed a bGWAS, including one isolate per clinical case and using ‘bacteraemia’ as the clinical endpoint (= ‘invasive infection’). If multiple isolates per clinical case had been collected, we chose isolates recovered from blood culture samples over isolates recovered from urine samples, as these caused the invasive infection. ‘Bacteraemia’ was defined as at least one positive *E. coli* blood culture, compared to ‘non-invasive UTI’ which was defined as at least one *E. coli*-positive urine sample, with no blood culture being tested positive for *E. coli* within 7 days.

**Fig. 3:**
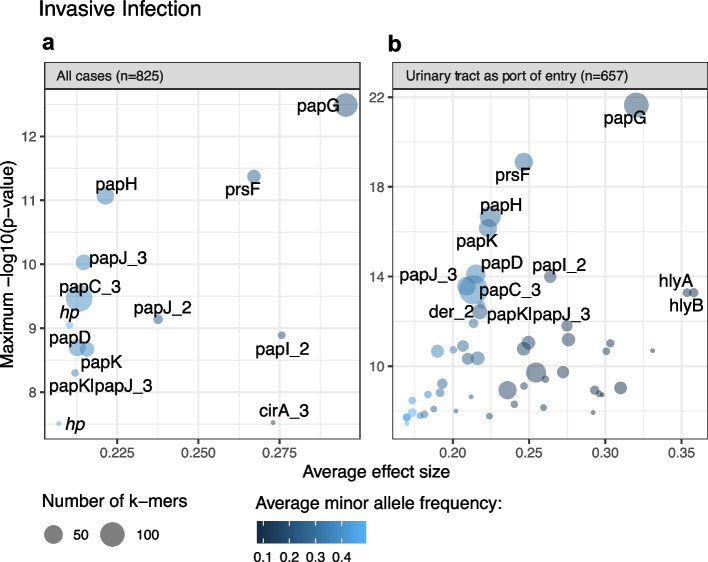
Significance level and average effect size of genes with mapping unitigs identified as significant in a bGWAS **a** including all clinical cases (*n* = 825) and **b** including cases for which the port of entry for bacteraemia could be assigned to the urinary tract. In **b**, only genes with a maximum − log10(*p*-value) > 11 are labelled. Genes with locus tags 100888-20_01189, 100033-19_04615, and 100033-19_04621 are labelled as papJ_2, papJ_3, and papI_2, respectively, as they were identified as such. Genes with the locus tags 100033-19_03736 and 100033-19_03452 are labelled as *hp* (= hypothetical protein)

We identified 13 genes to be significantly associated with invasive infection (Fig. 3a, Additional file 2: Table S1). The most significant hit corresponds to *papG* (89 unitigs (i.e. *k*mers of variable length)). Seventy of 89 of these *papG* unitigs had the highest ANI to the *papG* variant *papGII*, and 15/89 additional significant unitigs were equally similar to *papGII* and *papGIII*. Two of 89 unitigs had the highest similarity to *papGIII*, whereas 1 unitig each matched most closely to *papGI* and *papGIV* (Additional file 1: Fig. S6).

We identified nine additional genes of the *pap* operon to be associated with invasive infection: *prsF* (*papF*), *papH*, *papC*, *papK*, a gene annotated as *papJ|papK*, and genes with the locus tags *100888-20_01189* and *100033-19_04615* (both identified as *papJ* using blastp) and *100033-19_04621* (identified as *papI* using blastp). Furthermore, our GWAS identified unitigs mapping to the gene *cirA* as associated with invasive infection. CirA is embedded in the outer membrane, is postulated to participate in iron transport, and serves as a receptor for the colicins IA and IB [53]. For two genes (annotated as *100033-19_03452* and *100033-19_03736*), which also showed significant association with invasive infection, comparison to the EggNOG database identified NlpC/P60 family domains. Using NCBI blastn, both genes showed 100% sequence similarly with 100% query coverage to genes encoding C40 family peptidases and phage tail proteins (Additional file 2: Table S1).

Of the clinical cases which carried a *papGII*-positive *E. coli* strain, 56.7% (97/171) developed an invasive infection, whereas 25.1% (164/654) of *papGII*-negative clinical cases developed bacteraemia. Using *papGII* as a predictor for invasive infection had an accuracy of 71.2%, a sensitivity of 37.2%, a specificity of 86.9%, a positive predictive value of 56.7%, and a negative predictive value of 74.9%.

In a further analysis, we focused on UTI as the port of entry for sepsis. In a second bGWAS, we therefore excluded all invasive cases for which *E. coli* genomes were not available from isolates recovered from a blood culture and a urine sample, or where the *E. coli* isolate from the urine sample was phylogenetically distant to the blood culture isolate. In line with the previous bGWAS, the genes of the *pap*-operon are most significantly associated with invasive infection (Additional file 2: Table S2, Fig. 3b). In addition to the genes identified in our previous GWAS, we observed 41 more genes to be significantly associated with invasive infection. These include genes involved in basic cell metabolism, genes of unknown function, remnants of phages and other mobile genetic elements, and known *E. coli* virulence factors. These include *hlyA* (encoding an alpha-hemolysin [54]), *hlyB*, *hlyD* (involved in the hemolysin transport [55, 56]), and *cbtA* and *cbeA* (encoding the toxin and antitoxin part of a type IV toxin-antitoxin system, respectively [57, 58]).

In this smaller, more focused cohort, 41.3% (52/126) of cases which were infected with a *papGII*-positive *E. coli* strain developed an invasive infection, compared to 7.7% (41/531) of *papGII*-negative clinical cases. Using *papGII* as a single predictor for invasive infection had a sensitivity of 55.9%, a specificity of 86.9%, a positive predictive value of 41.3%, and a negative predictive value of 92.3%.

### *papGII* is associated with invasive infection when correcting for patient characteristics

To concurrently assess the impact of pathogen and patient-specific factors on the progression of UTI, we used a GLM with ‘bacteraemia’ as primary endpoint and ‘typical UTI symptoms’, ‘admission to ICU’, and ‘30 day all-cause mortality’ as secondary endpoints. As bacterial factors, we included *papGII* carriage, being the most prominent hit identified in our bGWASs. With the aim of identifying bacterial virulence and to correct for differences in AMR, we have additionally included phenotypically tested resistance against ceftriaxone as a bacterial factor in our model.

**Fig. 4:**
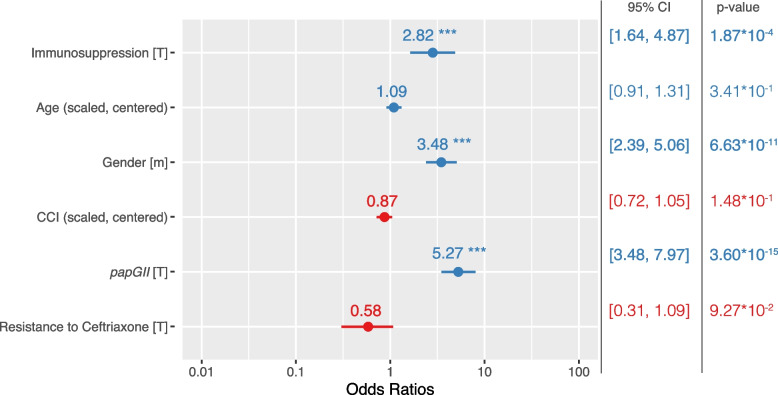
Odds ratio estimates with 95% confidence intervals for invasive infection using the generalised linear model (GLM). *n* = 751 complete observations with 210 events. CCI, Charlson Comorbidity Index; ***p-value < 0.001

Across all cases, *E. coli* strains encoding *papGII* were significantly more likely to be involved in invasive infection (OR 5.27, 95% CI = [3.48, 7.97], *p*-value < 0.001) (Fig. 4). We observed clinical cases with immunosuppression (OR 2.82, 95% CI = [1.64, 4.87], *p*-value < 0.001) and male patients (OR 3.48, 95% CI = [2.39, 5.06], *p*-value < 0.001) being more likely to be associated with invasive infection (Fig. 4). Moreover, when including host characteristics and phenotypic resistance to ceftriaxone as covariates in our bGWAS, *papG* was substantiated as being most significantly associated with invasive infection (Additional file 1: Fig. S7a, Additional file 2: Tables S1, S2).

Although not significant, and to a lesser extent, we also observe a tendency of *papGII* encoding *E. coli* strains to be more likely involved in (i) clinical cases with typical UTI symptoms (OR 1.44, 95% CI = [0.97, 2.13], *p*-value = 0.072) (Additional file 1: Fig. S7b) and (ii) clinical cases involving an ICU stay (OR 1.45, 95% CI = [0.96, 2.20], *p*-value = 0.078) (Additional file 1: Fig. S7c) than *E. coli* strains which do not encode *papGII*. Clinical cases with *papGII*-positive strains showed a significantly higher concentration of C-reactive Protein (CRP) (median = 115.6 mg/l, IQR = [47.8, 206.8] vs. median = 41.0 mg/l, IQR = [11.2, 113.5], *p*-value < 0.001, Mann–Whitney *U* test) (Additional file 1: Fig. S8a) and a higher leukocyte count in blood samples (median = 10.9 × 10^9^/l, IQR = [7.7, 14.4] vs. median = 9.1 × 10^9^/l, IQR = [6.5, 12.2], *p*-value < 0.001, Mann–Whitney *U* test), although the effect size in the latter is small (*r* = 0.15) (Additional file 1: Fig. S8b). Clinical cases with *papGII*-positive strains furthermore showed a higher bacterial cell count (median = 7194/μL, IQR = [1473, 12,914] vs. median = 5224/μL, IQR = [764, 11,218], *p*-value = 0.044, Mann–Whitney *U* test) (Additional file 1: Fig. S9a) and a higher ratio of leukocyte/bacterial cell counts in urine samples, compared to cases with a *papGII*-negative isolate (median = 0.19, IQR = [0.06, 0.68] vs. median = 0.10, IQR = [0.02, 0.45], *p*-value = 0.0018, Mann–Whitney *U* test) (Additional file 1: Fig. S9b). No difference in nitrite status (*p*-value = 0.07, Chi-squared test) (Additional file 1: Fig. S9c) nor in the ratio of erythrocytes/bacterial cell counts (*p*-value = 0.28, Mann–Whitney *U* test) (Additional file 1: Fig. S9d) was detected between urine samples with *papGII*-positive and *papGII*-negative *E. coli* strains.

We found no evidence of a *papGII*-specific association with 30-day mortality (*p*-value = 0.31) (Additional file 1: Fig. S7c). Patients 40 years and older were more often infected with *papGII*-negative strains (610/760, 80.3%) compared to patients younger than 40 years (44/65, 67.7%) (*p*-value = 0.03, Chi-squared test), whereas there was no evidence for varying *papGII* frequencies between male and female patients (*p*-value = 0.40, Chi-squared test) or between immunosuppressed and immunocompetent patients (*p*-value = 0.37, Chi-squared test) (Additional file 1: Fig. S10).

Older patients were less likely to experience typical UTI symptoms (OR 0.82, 95% CI = [0.70, 0.97], *p*-value = 0.018) (Additional file 1: Fig. S7a) and more likely to die within 30 days of sample collection (OR 2.54, 95% CI = [1.51, 4.28], *p*-value < 0.001). Patients with an increased CCI (OR 1.68, 95% CI = [1.28, 2.16], *p*-value < 0.001) were more likely to die within 30 days after the *E. coli* sample collection (Additional file 1: Fig. S7d).

Using the variables of our GLM including *papGII* as predictors for invasive infection yielded a mean accuracy of 74.7% (sd = 0.5%), a mean sensitivity of 25.9% (sd = 1.2%), a mean specificity of 93.6% (sd = 0.5%), a mean positive predictive value of 61.3% (sd = 2.4%), a mean negative predictive value of 76.5 (sd = 0.3%), and a mean AUROC of 73.6% (sd = 0.3%). Accuracy, negative predictive value, sensitivity, and AUROC decreased when omitting *papGII* as a predictor whereas specificity increased (*p*-value ≤ 0.01, paired Wilcoxon test) (Additional file 1: Fig. S7e).

### No evidence for *papGII* specific peak in MALDI-TOF mass spectra

Our results and previous studies highlight the importance of *papGII* in invasive *E. coli* infection. Its early detection in clinical diagnostics would be desirable and give evidence towards a severe progression of an ongoing UTI. MALDI-TOF MS is the most widely used tool for bacterial species identification in routine diagnostics, and we therefore aimed to assess whether there are *papGII*-specific signals in MALDI-TOF MS spectra. We did not identify any *papGII*-specific signal, nor a peak being associated with the absence of any *papG* variant (Additional file 1: Fig. S11).

As *papGII* presence is associated with the ExPEC phylogroups B2-1, B2-2, D1, D2, D3, and F, we further investigated whether the *E. coli* phylogroups can be distinguished by MALDI-TOF MS. We identified a mass shift from around 9711 to 9739 Da, which uniquely distinguishes the phylogroups B2-1, B2-2, and F from all other phylogroups (Additional file 1: Fig. S12). This mass shift has previously been identified as two different mass alleles of the acid stress chaperone HdeA [59]. From MALDI-TOF mass spectra (Additional file 1: Fig. S12) and predicted from genomic data (Additional file 1: Fig. S13), we observe the HdeA mass allele at 9712 Da in the ExPEC associated phylogroups B2-1, B2-2, and F and the mass allele at 9740 Da in the phylogroups A and B1 which are associated with a colonisation clinical phenotype. However, the ExPEC associated phylogroups D1, which also carries *papGII*, and D3 also encoded HdeA with a mass of 9740 Da. Strains of the phylogroup D2 were observed to encode HdeA of either 9712 Da or 9740 Da (Additional file 1: Fig. S12 and Fig. S13).

### *papGII* can be identified by qPCR directly from urine samples

As an alternative diagnostic method, we designed and validated qPCR primers and probes to detect *E. coli* strains encoding *papGII*. After verifying the functionality of our primers and probes (Additional file 1: Fig. S14-S18, Additional file 1: Supplementary PCR Data, Additional file 2: Table S3), we prospectively screened 1657 urine samples at two healthcare centres (*n* = 529 at centre 1 and *n* = 1128 at centre 2).

Of the 261 samples that tested culture positive for *E. coli* in centre 1 in the routine diagnostic, a *gapC* signal was detected in 98.8% (258/261) using our PCR assay. A *gapC* signal was further recorded in 36.2% (97/268) of samples, for which no *E. coli* was reported in clinical routine diagnostics, possibly reflecting either the increased sensitivity of our qPCR assay compared to culture-based detection and reporting of *E. coli*, or unspecific amplification. *papC* was detected in 267/529 samples, 63.7% (170/267) of which were culture positive for *E. coli*. Finally, *papGII* was detected in 40/529 urine samples which were all *E. coli* culture-positive. We further evaluated which clinical cases for which *E. coli* had been cultured in routine diagnostics (*n* = 244, excluding multiple samples from the same patient) developed an invasive infection. An *E. coli*-positive blood culture was recorded in 8.3% (3/36) of cases for which a *papGII*-encoding *E. coli* had been identified in the urine sample, compared to 4.3% (9/208) of cases for which an *E. coli* with no *papGII* had been identified in the urine sample (*p*-value 0.54, Chi-squared test).

In comparison with centre 1 where all urine samples were prospectively screened, we exclusively screened culture-positive *E. coli* isolates at centre 2 (*n* = 1106). When including one sample per patient only (*n* = 871), we detected *papC* in 33.8% of cases (293/868, 3 measurements were excluded as the positive control (*rpoD*) did not test positive) and *papGII* in 16.4% of cases (143/871). Patients with a *papGII*-positive strain developed more frequently an invasive infection than patients with a *papGII*-negative strain (11.9% (17/143) vs. 6.5% (47/728), *p*-value = 0.036, chi-squared test).

## Discussion

In this study, we concurrently analysed the bacterial genomic factors and patient characteristics and highlighted the importance of *papGII* in invasive UTI . Consistent with previous studies, we identified *papGII* in globally successful UPEC lineages [13].

*papGII* often co-occurred with *iuc* and rarely occurred in strains which were resistant to ceftriaxone except within the globally disseminating ESBL UPEC lineage ST131. We observed over 60% of our strains and over 95% of our ExPEC phylogroup strains carrying genes which encode for polysaccharide capsules. This is a substantially larger fraction than has previously been reported in *E. coli* RefSeq sequences where less than 25% of assemblies carried the *kpc* locus [31]. Capsular polysaccharides facilitate Gram-negative bacteria to evade the innate host immune response [60]. For example, the *E. coli* capsules K1 and K5 have been shown to do so by molecular mimicry because identical polysaccharides are present on human cells [61]. The increased proportion of polysaccharide capsules in our collection gives further evidence on the importance of capsules for *E. coli* to infect the human urinary tract. However, neither the data collected in this study nor those from previous studies assessing *E. coli* bloodstream infection [13, 17] suggested an association of these capsule loci with severe progression of UTI to invasive infection.

In a subset of samples, we identified the *E. coli* strain isolated from the urine sample being unrelated to the *E. coli* strain isolated from the bloodstream from the same clinical case. Possible explanations for this observation are (i) a multi-strain infection of the urinary tract or (ii) a different port of entry to the bloodstream than the urinary tract. In a continuous analysis and in line with previous studies [62, 63], we identified strains of different phylogroups isolated from the same urine sample which supports explanation (i). However, as we examined multiple colony picks from the same urine sample only for a small number of cases, further studies are required to assess the within-host *E. coli* strain diversity infecting the urinary tract and their dynamics in disease progression. Infections caused by multiple strains of the same species pose a challenge in diagnostics, as they are not systematically screened for using culture-based methods, relying on a pure culture often originating from a single colony pick.

In line with a previous study [13], we identified genes of the *pap* operon, and most significantly *papGII*, as being associated with invasive infection in our bacterial GWASs. The gene *papGII* encodes one of multiple variants (PapGI–V) of the adhesin tips of Pap pili, and binds to the globoseries of glycosphingolipids, more specifically Gb5 (GalNAcα1-3-GalNAc3Galα1-4Galβ1-4GlcCer) [13, 64] of human uroepithelial and kidney cells [65, 66] and can modulate the host immune response [67]. A previous study suggests that virulent UPEC lineages emerged after the independent horizontal acquisition of pathogenicity islands encoding *papGII* [13].

Factors associated with horizontal gene transfer (HGT) (e.g. phage/prophage-associated proteins) and *E. coli* toxins were identified as associated with invasive infection in our bGWASs, although less significantly than *papGII*. These genes could potentially originate from the horizontal acquisition of the pathogenicity island encoding the *papGII*, which carries varying gene content, occasionally including these toxins (*hlyA*, *hlyB*, *hlyD* on PAI types II, III and VI), as well as phage gene remnants and Insertion Sequence (IS) elements [13].

The crucial role of *papGII* in invasive infections is further supported by our GLM, where we concurrently correct our analysis for important patient characteristics as well as resistance against ceftriaxone. Correcting for patient characteristics is a crucial step to estimate bacterial virulence. Without such analyses, it is not possible to assess whether an observed increase in virulence is caused by a bacterial genetic factor or might arise from patient confounding factors. Our analysis also identified the higher likelihood of male patients developing invasive infection, which can presumably be explained by the increased occurrence of uncomplicated UTI in female patients [2]. In our GLM, we did not consider other ports of entry for sepsis than the urinary tract, which certainly is a limitation of the current study as this might influence the clinical outcomes. The urinary tract has previously been determined as the entry point in 50–60% of *E. coli* sepsis [17]. As *papGII* binds to human uroepithelial and kidney cells [66, 67], its impact on patient outcome might be even larger when exclusively examining patients with urosepsis, compared to all bloodstream infections.

We assessed the predictive value for invasive infection when using either (a) *papGII*, (b) the variables in our GLM excluding *papGII*, or (c) the variables in our GLM including *papGII* as input variables in a simple classifier. As expected for a complex clinical outcome such as invasive infection, did none of the three approaches yield high enough predictive values to be applicable as a diagnostic test for invasive infection. Despite a relatively low sensitivity, we observed the highest accuracy when including the variables in our GLM including *papGII* as predicting variables. We hypothesise that the performance of classifiers predicting invasiveness will dramatically increase when using a larger input dataset, using a more specific patient cohort (e.g. focussing on the urinary tract as port of entry for sepsis) and by using more elaborate statistical approaches.

In this study, we classified clinical cases into ‘invasive’ (positive blood culture) and ‘non-invasive’ (positive urine culture, no positive blood culture) infections based on microbiological endpoints. This classification is not always consistent with a clinical assessment. A more refined classification of cases as well as considering antimicrobial treatments and co-morbidities in more detail would further improve the risk assessment of bacterial and patient factors. A more detailed analysis should further consider the primary reason for hospitalisation, whether the UTI was acquired in the hospital or not and which surgical/non-surgical interventions patients underwent during their hospital stay.

We examined whether there are MALDI-TOF MS peaks which are specific for *E. coli* strains carrying *papGII* and which could be used to detect these virulent UPEC clones in clinical routine diagnostics. The *papGII* protein weighs 37,667 Da [68] and lies beyond the mass range of MALDI-TOF MS devices routinely used for bacterial species identification. Unfortunately, no alternative peak was observed which could serve as a surrogate marker. We did, however, observe a previously described mass shift [69] of the acid stress chaperone *HdeA* which is specific for the ExPEC phylogroups B2-1, B2-2, and F but fails to discriminate the ExPEC phylogroup D1-3 from the carriage associated phylogroups A, B1, and C. Although in this simple analysis no single signal was observed which unambiguously identifies *papGII*-positive strains from MALDI-TOF mass spectra, more elaborate statistical analysis might still reveal a combination of peaks or intensity patterns allowing for the identification of such strains.

As an alternative approach to screen patient samples for *E. coli*, the *pap* operon and *papGII*, we designed qPCR primers and probes for three targets and tested their performance when applied directly to urine samples. The main advantage of this method is the short turnaround time, which allows results to be obtained a few hours after sampling as the cultivation step can be omitted. An additional advantage of omitting the cultivation step is avoiding a bias toward the most dominant strain in a multi-strain infection and thereby being less prone to miss the invasive potential of a sub-dominant strain.

For the primer pairs for the *E. coli* marker *gapC* and the pap-operon marker *papC*, amplifications were recorded in multiple (97/268 each) samples for which no positive *E. coli* culture was reported. These could correspond to non-specific amplifications or, as we hypothesise, indicate an increased sensitivity of qPCR compared to culture-based detection and reporting of *E. coli*. To be applicable in routine diagnostics, this method would have to be further validated, including samples with other *Enterobacteriaceae*.

In both centres where we prospectively screened *E. coli* strains infecting the urinary tract, we observed the relative frequency of invasive infection to be > 1.8-fold higher for *papGII*-positive strains compared to *papGII*-negative strains which is in line with our first cohort included in our bGWAS. These findings from an independently collected patient cohort further substantiate the importance of *papGII* in invasive infections, which we have previously highlighted by analysing bacterial genomic data and important patient characteristics.

## Conclusions

This study builds on previous work identifying *papGII* with invasive infection and shows that it is a major risk factor for progression from UTI to bacteraemia that has diagnostic potential. It exemplifies the importance of including patient data to assess the potential virulence of a bacterial pathogen.

### Supplementary Information

**Additional file 1:** **Fig. S1.** Distribution of *E. coli *phylogroups (left) and Sequence Types (ST) (right) in male (*n*=251) (upper row) and female (*n*=574) (lower row) patients. **Fig. S2.** Distribution of *E. coli* phylogroups (left) and Sequence Types (ST) (right) in in female patients (*n*=574) younger than 40 years (*n*=52) (upper row) and older than 40 years (*n*=522) (lower row) patients. **Fig. S3.** Distribution of *E. coli *phylogroups (left) and Sequence Types (ST) (right) in invasive infections (*n*=261) (upper row) and non-invasive infections (*n*=574) (lower row) patients. **Fig. S4.** Core genome phylogeny of 825 *E. coli *strains. Columns represent (from left to right): the assigned phylogroup, the sequence type, phenotypic resistance against ceftriaxone, meropenem, fosfomycin, nitrofurantoin and ciprofloxacin. **Fig. S5. **Within host genetic diversity of *E. coli* strains isolated from the same clinical cases. a: core genome phylogeny of *E. coli* strains (*n*=225), isolated from the same clinical case (*n*=106), coloured by phylogroup. The numbers correspond to the case identifier and strains were only labelled, if they exhibited < 99.9% Average Nucleotide Identity to the strain isolated from the same clinical case. b: *papG *variant encoded by isolates which exhibited < 99.9% Average Nucleotide Identity to the strain isolated from the same clinical case. c: Average Nucleotide Identity of strains isolated from case 3. d: SNV of 10 picked isolates from three cases, either from urine or blood culture samples. **Fig. S6.** Average Nucleotide Identity values for unitigs identified in our bGWAS and mapping to *papG* (X-axis) and the reference sequences for the five *papG* variants (Y-axis). **Fig. S7.** a: Significance level and average effect size of genes with mapping unitigs identified as significant in a bGWAS including all clinical cases (*n*=751 complete observations) (right) and including cases for which the port of entry for bacteraemia could be assigned to the urinary tract (*n*=612 complete observations) (right). In the right figure only genes with a maximum -log10(*p*-value) > 11 are labelled. Genes with locus tags 100888-20_01189, 100033-19_04615 and 100033-19_04621 are labelled as papJ_2, papJ_3 and papI_2, respectively, as they were identified as such. The gene with the locus tags 100033-19_03452 are labelled as ‘*hp*’ (= hypothetical protein); Odds ratio estimates with 95% confidence intervals for b: Typical urinary tract infection symptoms (*n* = 717 complete observations with 213 events); c: Admission to the intensive care unit (*n* = 751 complete observations with 172 events); d: 30-day all cause mortality (*n* = 749 complete observations with 45 events); using the generalised linear model (GLM). e: Performance of GLM classifiers using ‘Invasive disease’ as outcome variable and the same dataset as and variables as in the GLM as input (751 complete observations with 210 events), either including the presence of papGII as a predictor or not. Error bars indicate the standard deviation and the means were compared using paired Wilcoxon tests. OR = odds ratio; CI = confidence interval; CCI = Charlson Comorbidity Index; ‘AUROC’: area under the receiver operating curve; ‘NPV’: negative predictive value; ‘PPV’: positive predictive value; ‘ns’ = not significant; ‘*’ = *p*-value < 0.05; ‘**’ = *p*-value < 0.01; ‘***’ = *p*-value < 0.001. **Fig. S8.** C-reactive protein concentration (a) and leucocyte count (b) measured in blood samples of cases, for which a *papGII *positive or a *papGII* negative *E. coli *strain was isolated from a urine or a blood culture samples. Leucocyte counts were measured on the day the urine / blood culture samples were taken. **Fig. S9.** (a) Bacterial cell count, (b), leucocyte count divided by bacterial cell count (c) nitrite status and (d) erythrocyte count divided by bacterial cell count measured in urine samples of cases, for which a *papGII* positive or a *papGII *negative *E. coli* strain was isolated from a urine or a blood culture samples. **Fig. S10.** Relative occurrence of *papGII *in isolates from patients younger vs. older than 40 years, in isolates from male vs. female patients and in isolates from patients which were immunosuppressed vs. patients which were not immunosuppressed. **Fig. S11.** Occurrence of MALDI-TOF mass peaks in spectra acquired from *E. coli* strains encoding no *papG* gene, encoding a *papG* variant other than* papGII* and encoding *papGII*. ‘Occurrence’ refers to the percentage of spectra per group in which a peak was detected. Each strain was measured in quadruplicate either on a Microflex Biotyper device, or an Axmina Confidence device. Masses are only depicted if detected in > 30% or < 25% of spectra for one or more of the groups. **Fig. S12.** Occurrence of MALDI-TOF mass peaks in spectra acquired from *E. coli* strains of different phylogroups. ‘Occurrence’ refers to the percentage of spectra per group in which a peak was detected. Each strain was measured in quadruplicate either on a Microflex Biotyper device, or an Axmina Confidence device. Phylogroups for which less than five strains were available (E1, E2 and G) were excluded from the plot. Masses are only depicted if detected in > 50% or < 25% of spectra for one or more of the groups. **Fig. S13.** Core genome phylogeny of the* E. coli *strains collected for this study (one strain per clinical case, *n*=825). Phylogroup assignment, Sequence Type (ST) (eight most frequent ones coloured, more rare STs in grey), *papG* variant, mass of HdeA, predicted from the amino acid sequence. **Fig. S14.** Results of the endpoint PCR assay (a) to test the functionality of the primers designed at centre 1. This also includes tests for the cross reactivity between *papGII *and *papGIII *primers. (b) to test the functionality of the *rpoD* primers designed at centre 2. **Fig. S15.** Evaluating the efficiency of primers and probes used in our qPCR assay (a) qPCR standard curves and values for the primer pairs gapC_2, papC_1, uidA and papGII_2 tested at centre 1. Each measurement was performed in triplicate. (b) Amplification plots for the two *rpoD* probes designed at centre 2. Measurements performed in quadruplicate. **Fig. S16.** Variants of primer and probe sequences detected in our genome collection (*n*=1,076). Sequences used in the qPCR assay are indicated in blue and alternative variants detected in the genomes are depicted in black. Variants were called using the variantcaller Freebayes via snippy and using a minimum coverage of 20x. **Fig. S17.** (a) Efficiency of the primer pairs in the single reaction (blue) and in a triplex reaction (orange) for the primers used at center 1 (*gapC*, *papC* and *papGII*). (b) Amplification curves of primers used at center 2: *rpoD* and *papGII* in duplex reactions and of *papGII* in a triplex reaction with *rpoD* and *papC*. **Fig. S18.** Comparison of the Ct-value yielded when processing urine pellets (*n*=24) using the QIAamp DNA Mini Kit and after boiling for 10 minutes. **Supplementary Methods.** Endpoint PCR, Quantitative PCR, Multiplexing the qPCR, Applying qPCR assay directly to urine samples, Screening of patient samples. **Supplementary PCR Data:** Evaluation of primer functionality.

**Additional file 2:** **Table S1a.** Significant gene hits identified to be associated with invasive infection including 825* E. coli* strains using pyseer. **Table S1b.** Significant gene hits identified to be associated with invasive infection including 751 *E. coli *strains and using important host characteristics as covariates. **Table S2a.** Significant gene hits identified to be associated with invasive infection including 657 *E. coli *strains using pyseer. **Table S2b.** Significant gene hits identified to be associated with invasive infection including 612 *E. coli *strains and using important host characteristics as covariates. **Table S3a.** qPCR evaluation metrics recorded for eight different primer pairs and genomic DNA which was previously extracted using the QIAamp DNA Mini Kit. **Table S3b.** qPCR evaluation metrics recorded for four different primer pairs and using urine samples which were boiled.

## Data Availability

The raw reads acquired for this study have been submitted to EBI ENA and are publicly available (Project Accession PRJEB55855, https://www.ebi.ac.uk/ena/browser/view/pRJEB55855) [70]. The MALDI-TOF mass spectra acquired for this study can be accessed via the OpenScienceFoundation (https://osf.io/vmqc5/) [47]. All code which was used to visualise bacterial data analysed in this study is available on GitHub (https://github.com/acuenod111/UPEC) [46].

## Abbreviations

AMR: Antimicrobial resistance
ANI: Average nucleotide identity
bGWAS: Bacterial genome-wide association study
CCI: Charlson comorbidity index
COS: Columbia sheep blood
CRP: C-reactive protein
ESBL: Extended-spectrum β-lactamase
GLM: Generalised linear model
ICU: Intensive care unit
IQR: Interquartile range
MALDI-TOF MS: Matrix-assisted laser desorption/ionisation time of flight
MLST: Multi-locus sequence typing
MS: Mass spectrometry
OR: Odds ratio
PAP: Pyelonephritis-associated pili
PCR: Polymerase chain reaction
SNV: Single nucleotide variant
ST: Multi-locus sequence type
UPEC: Uropathogenic *Escherichia coli*
UTI: Urinary tract infection
