# Supplementary material for: Bacterial genome-wide association study substantiates papGII of Escherichia coli as a major risk factor for urosepsis
Source: Genome Med. 2023 Oct 30;15:89. doi: 10.1186/s13073-023-01243-x (PMC10614358; doi:10.1186/s13073-023-01243-x)
Supplement: Supplementary file 1 — Additional file 1: Fig. S1. Distribution of E. coli phylogroups (left) and Sequence Types (ST) (right) in male (n=251) (upper row) and female (n=574) (lower row) patients. Fig. S2. Distribution of E. coli phylogroups (left) and Sequence Types (ST) (right) in in female patients (n=574) younger than 40 years (n=52) (upper row) and older than 40 years (n=522) (lower row) patients. Fig. S3. Distribution of E. coli phylogroups (left) and Sequence Types (ST) (right) in invasive infections (n=261) (upper row) and non-invasive infections (n=574) (lower row) patients. Fig. S4. Core genome phylogeny of 825 E. coli strains. Columns represent (from left to right): the assigned phylogroup, the sequence type, phenotypic resistance against ceftriaxone, meropenem, fosfomycin, nitrofurantoin and ciprofloxacin. Fig. S5. Within host genetic diversity of E. coli strains isolated from the same clinical cases. a: core genome phylogeny of E. coli strains (n=225), isolated from the same clinical case (n=106), coloured by phylogroup. The numbers correspond to the case identifier and strains were only labelled, if they exhibited < 99.9% Average Nucleotide Identity to the strain isolated from the same clinical case. b: papG variant encoded by isolates which exhibited < 99.9% Average Nucleotide Identity to the strain isolated from the same clinical case. c: Average Nucleotide Identity of strains isolated from case 3. d: SNV of 10 picked isolates from three cases, either from urine or blood culture samples. Fig. S6. Average Nucleotide Identity values for unitigs identified in our bGWAS and mapping to papG (X-axis) and the reference sequences for the five papG variants (Y-axis). Fig. S7. a: Significance level and average effect size of genes with mapping unitigs identified as significant in a bGWAS including all clinical cases (n=751 complete observations) (right) and including cases for which the port of entry for bacteraemia could be assigned to the urinary tract (n=612 complete observations) [file 13073_2023_1243_MOESM1_ESM.pdf]

Gender

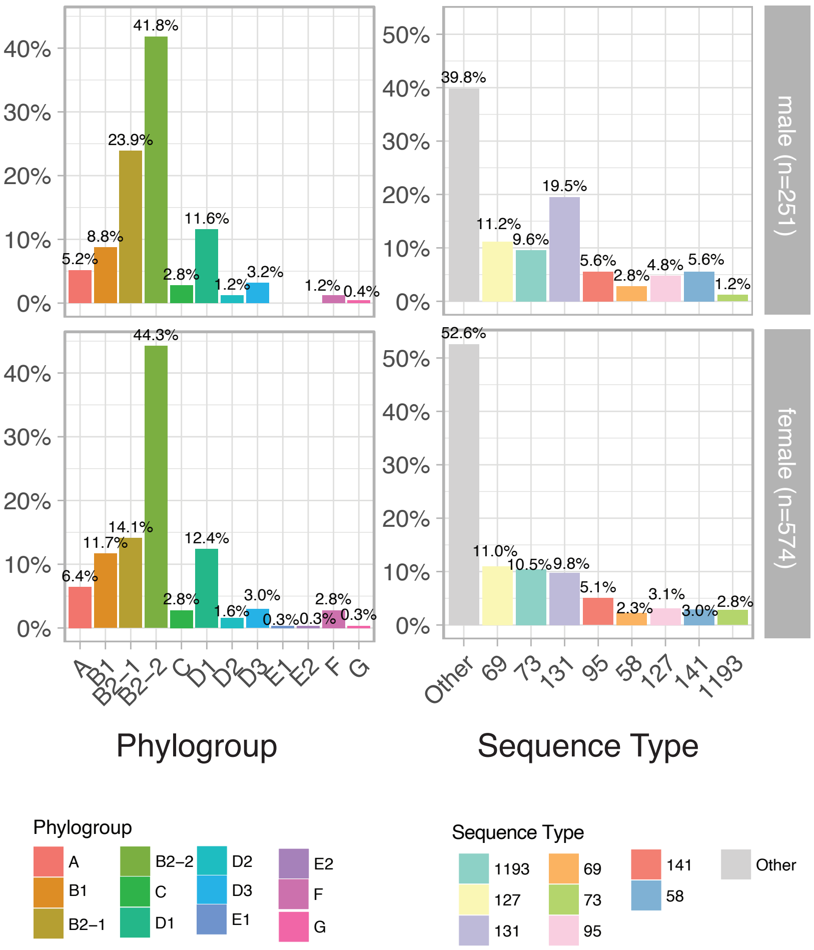

Figure S1: Distribution of *E. coli* phylogroups (left) and Sequence Types (ST) (right) in male (n=251) (upper row) and female (n=574) (lower row) patients.

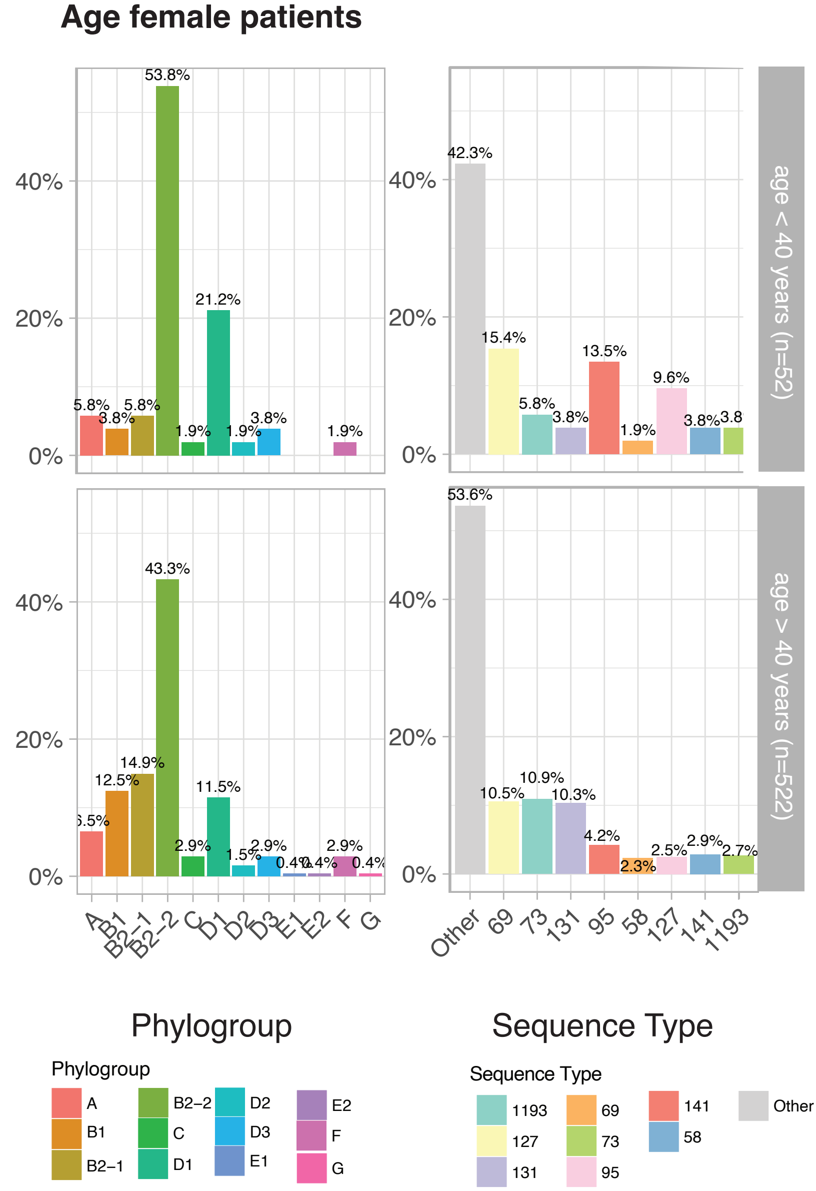

Figure S2: Distribution of *E. coli* phylogroups (left) and Sequence Types (ST) (right) in in female patients (n=574) younger than 40 years (n=52) (upper row) and older than 40 years (n=522) (lower row) patients.

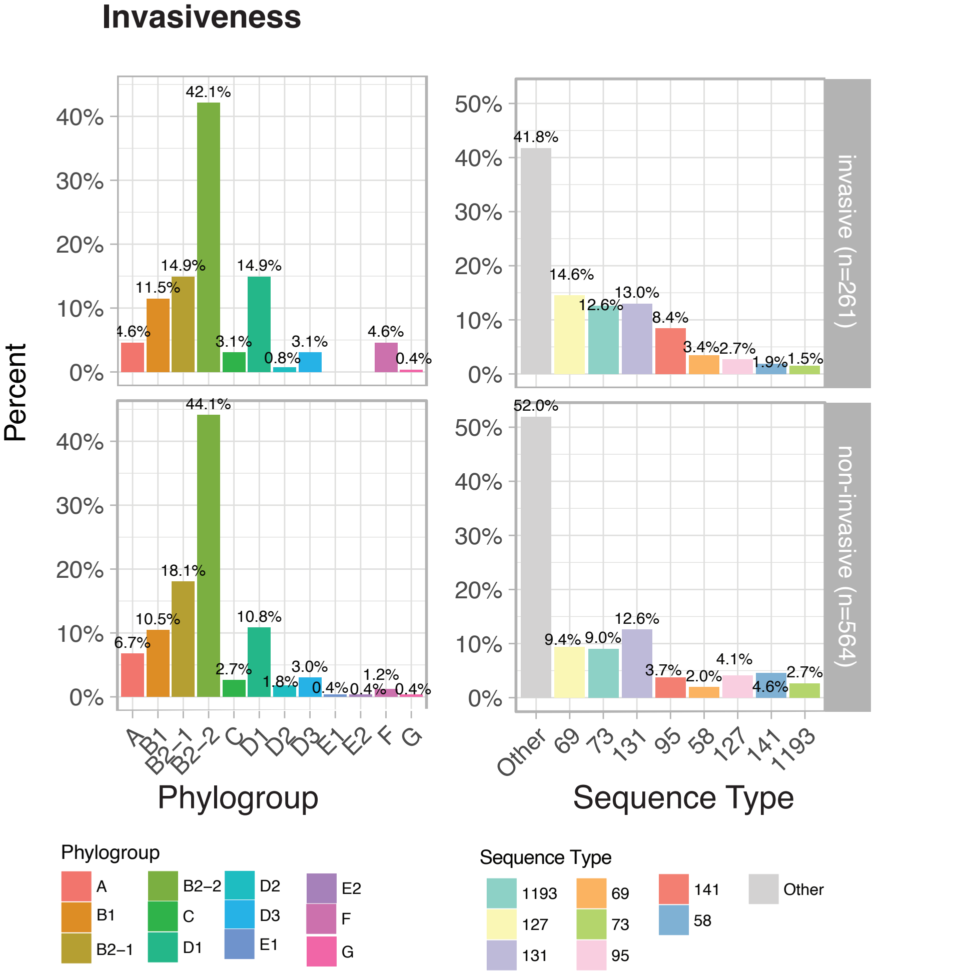

Figure S3: Distribution of *E. coli* phylogroups (left) and Sequence Types (ST) (right) in invasive infections (n=261) (upper row) and non-invasive infections (n=574) (lower row) patients.

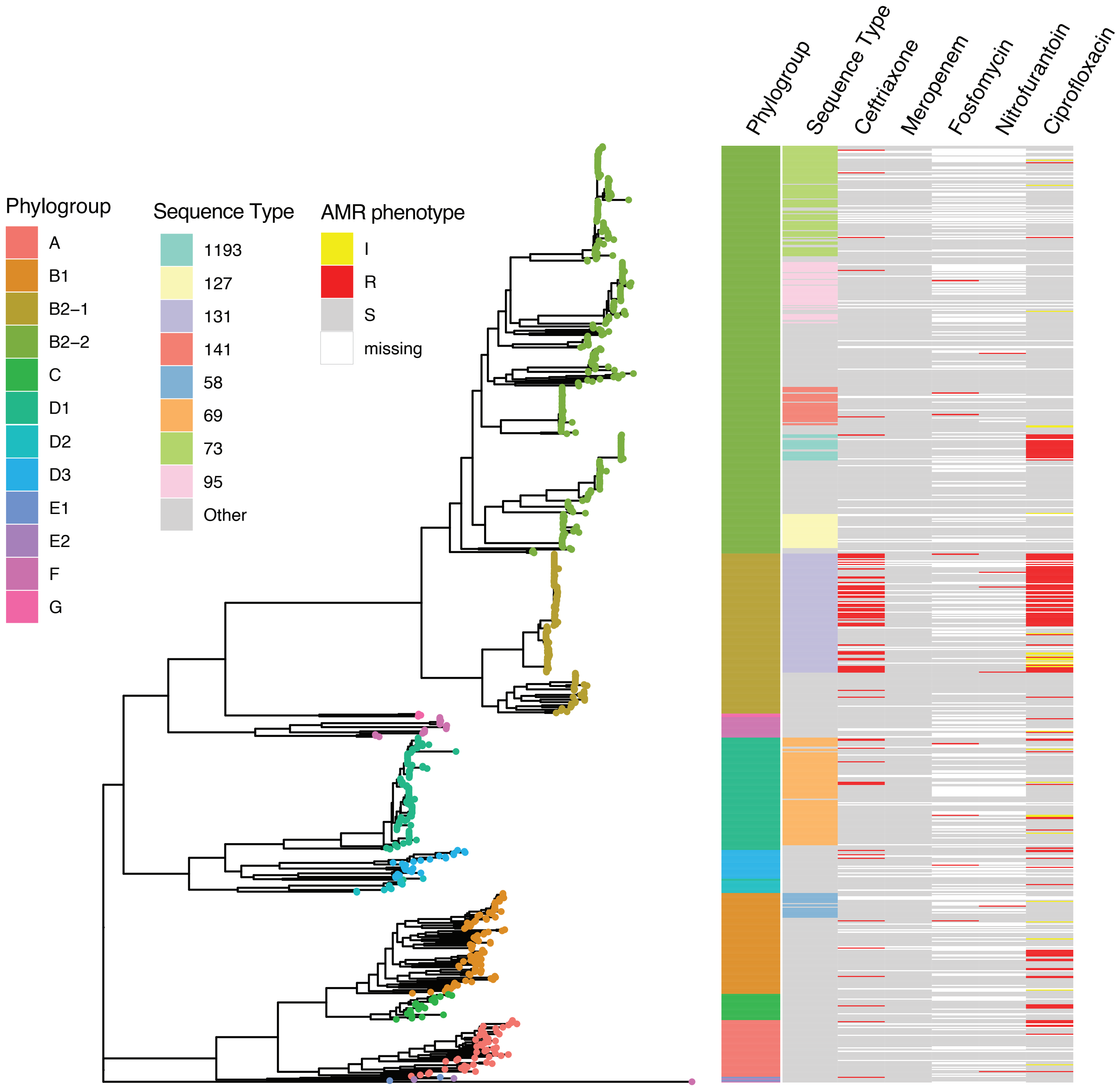

Figure S4: Core genome phylogeny of 825 *E. coli* strains. Columns represent (from left to right): the assigned phylogroup, the sequence type, phenotypic resistance against ceftriaxone, meropenem, fosfomycin, nitrofurantoin and ciprofloxacin.

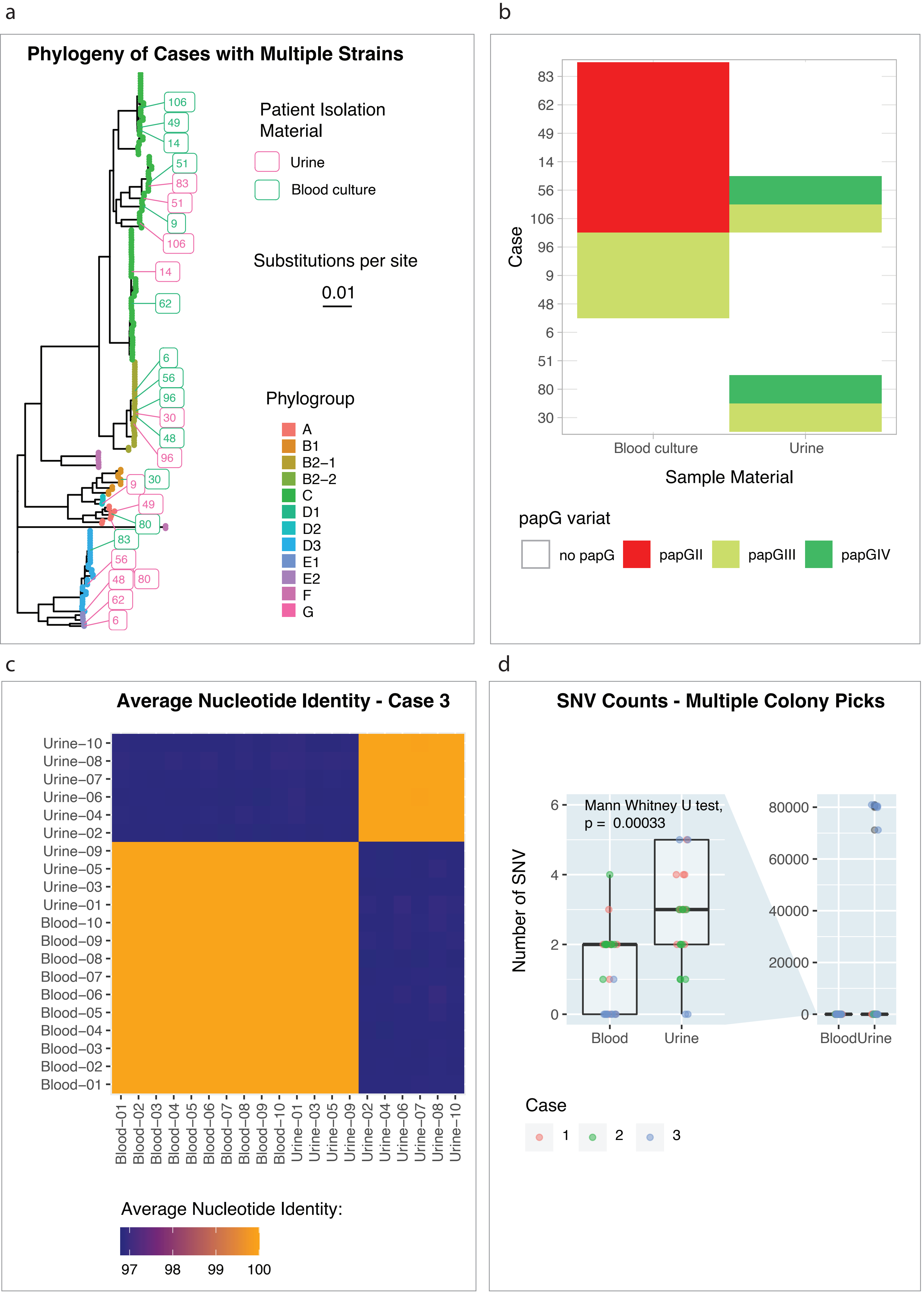

Figure S5: Within host genetic diversity of *E. coli* strains isolated from the same clinical cases. **a:** core genome phylogeny of *E. coli* strains (n=225), isolated from the same clinical case (n=106), colored by phylogroup. The numbers correspond to the case identifier and strains were only labelled, if they exhibited < 99.9% Average Nucleotide Identity to the strain isolated from the same clinical case. **b:** papG variant encoded by isolates which exhibited < 99.9% Average Nucleotide Identity to the strain isolated from the same clinical case. **c:** Average Nucleotide Identity of strains isolated from case 3. **d:** SNV of 10 picked isolates from three cases, either from urine or blood culture samples.

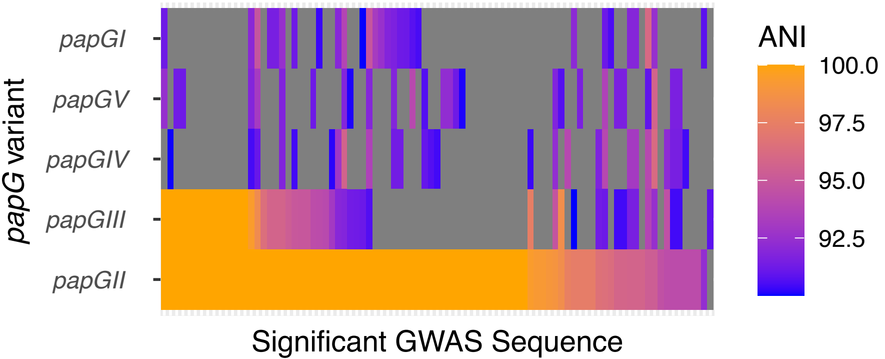

Figure S6: Average Nucleotide Identity values for unitigs identified in our bGWAS and mapping to *papG* (X-axis) and the reference sequences for the five *papG* variants (Y-axis).

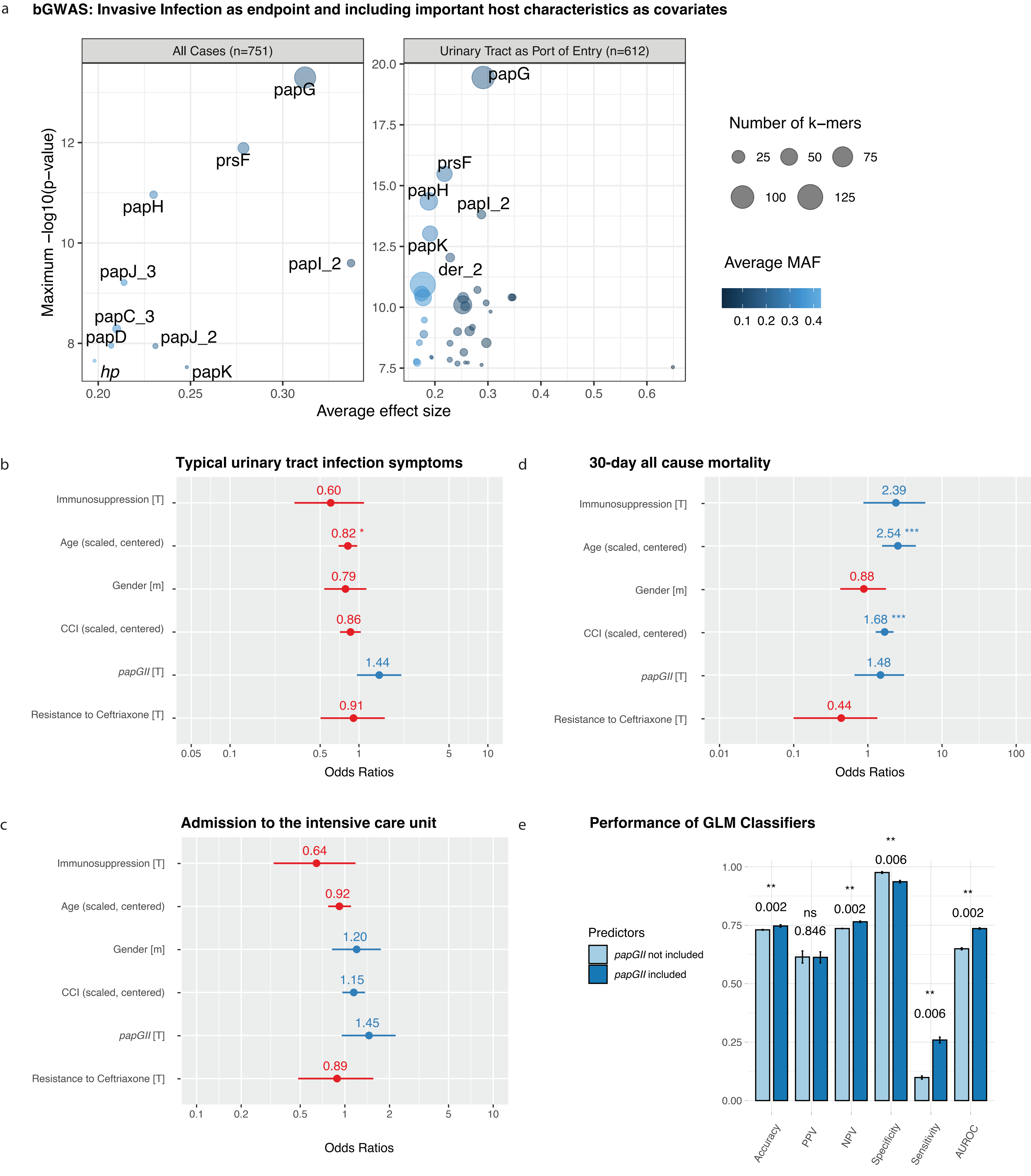

Figure S7: **a:** Significance level and average effect size of genes with mapping unitigs identified as significant in a bGWAS including all clinical cases (n=751 complete observations) (right) and including cases for which the port of entry for bacteraemia could be assigned to the urinary tract (n=612 complete observations) (right). In the right figure only genes with a maximum -log10(p-value) > 11 are labelled. Genes with locus tags 100888-20_01189, 100033-19_04615 and 100033-19_04621 are labelled as papJ_2, papJ_3 and papI_2, respectively, as they were identified as such. The gene with the locus tags 100033-19_03452 are labelled as 'hp' (= hypothetical protein); Odds ratio estimates with 95% confidence intervals for **b**: Typical urinary tract infection symptoms (n = 717 complete observations with 213 events); **c**: Admission to the intensive care unit (n = 751 complete observations with 172 events); **d**: 30-day all cause mortality (n = 749 complete observations with 45 events); using the generalised linear model (GLM). **e**: Performance of GLM classifiers using 'Invasive disease' as outcome variable and the same dataset as and variables as in the GLM as input (751 complete observations with 210 events), either including the presence of *papGII* as a predictor or not. Error bars indicate the standard deviation and the means were compared using paired Wilcoxon tests. OR = odds ratio; CI = confidence interval; CCI = Charlson Comorbidity Index; 'AUROC': area under the receiver operating curve; 'NPV': negative predictive value; 'PPV': positive predictive value; 'ns' = not significant; '*' = p-value < 0.05; '**' = p-value < 0.01; '***' = p-value < 0.001

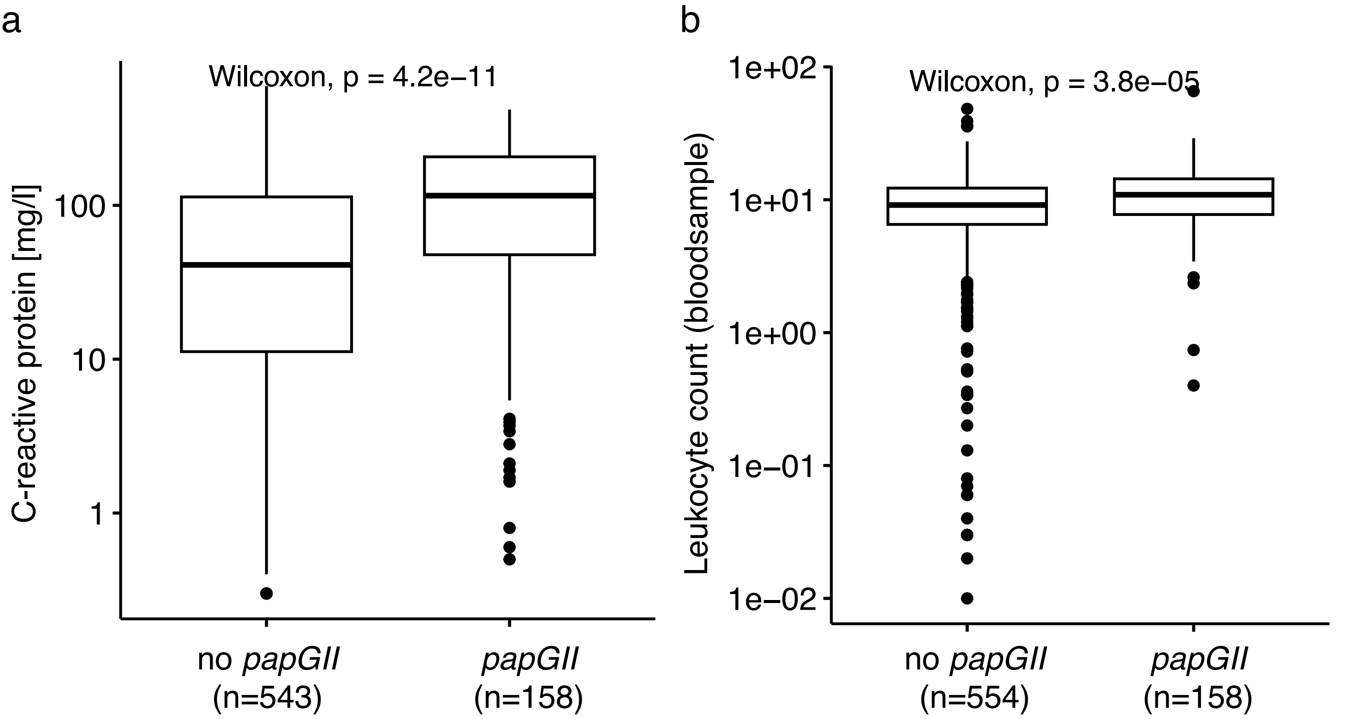

Figure S8: C-reactive protein concentration (**a**) and leucocyte count (**b**) measured in blood samples of cases, for which a *papGII* positive or a *papGII* negative *E. coli* strain was isolated from a urine or a blood culture samples. Leucocyte counts were measured on the day the urine / blood-culture samples were taken.

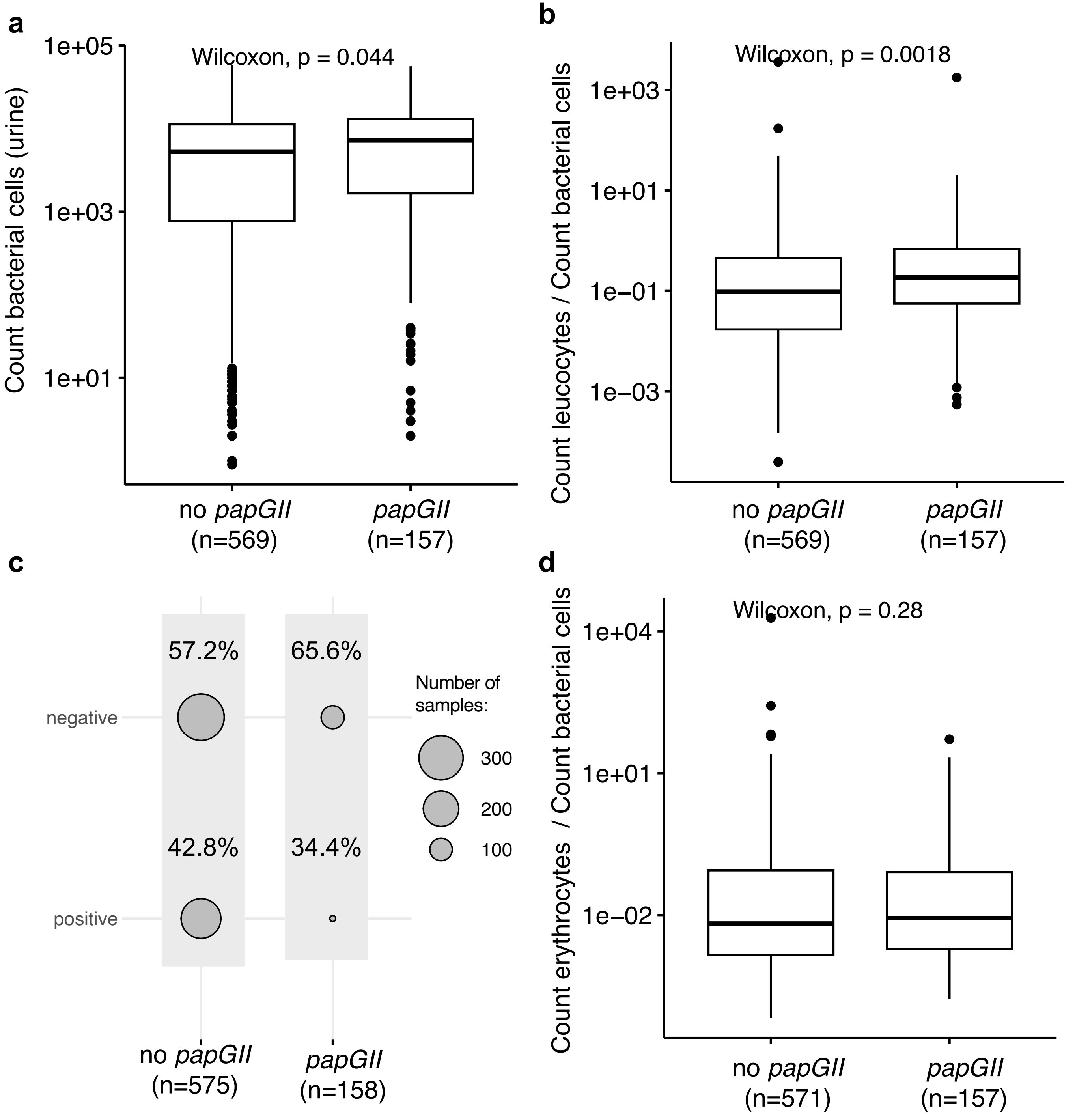

Figure S9: (**a**) Bacterial cell count, (**b**), leucocyte count divided by bacterial cell count (**c**) nitrite status and (**d**) erythrocyte count divided by bacterial cell count measured in urine samples of cases, for which a *papGII* positive or a *papGII* negative *E. coli* strain was isolated from a urine or a blood culture samples.

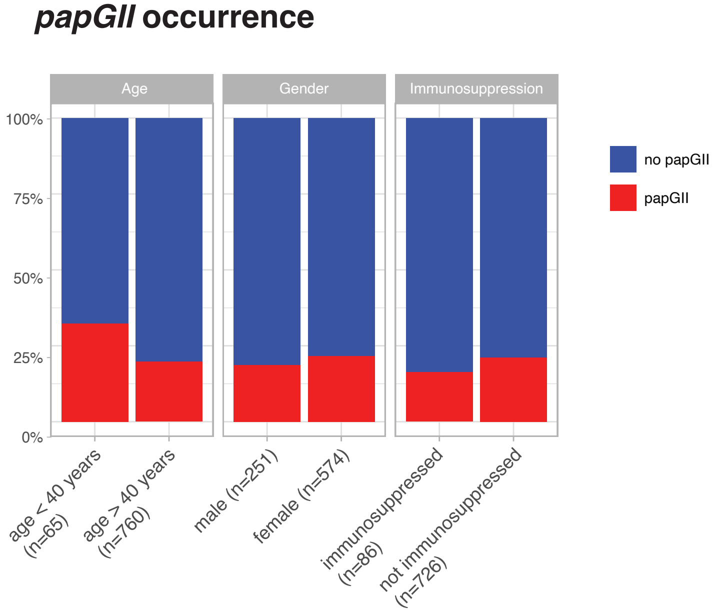

Figure S10: Relative occurrence of *papGII* in isolates from patients younger vs. older than 40 years, in isolates from male vs. female patients and in isolates from patients which were immunosuppressed vs. patients which were not immunosuppressed.

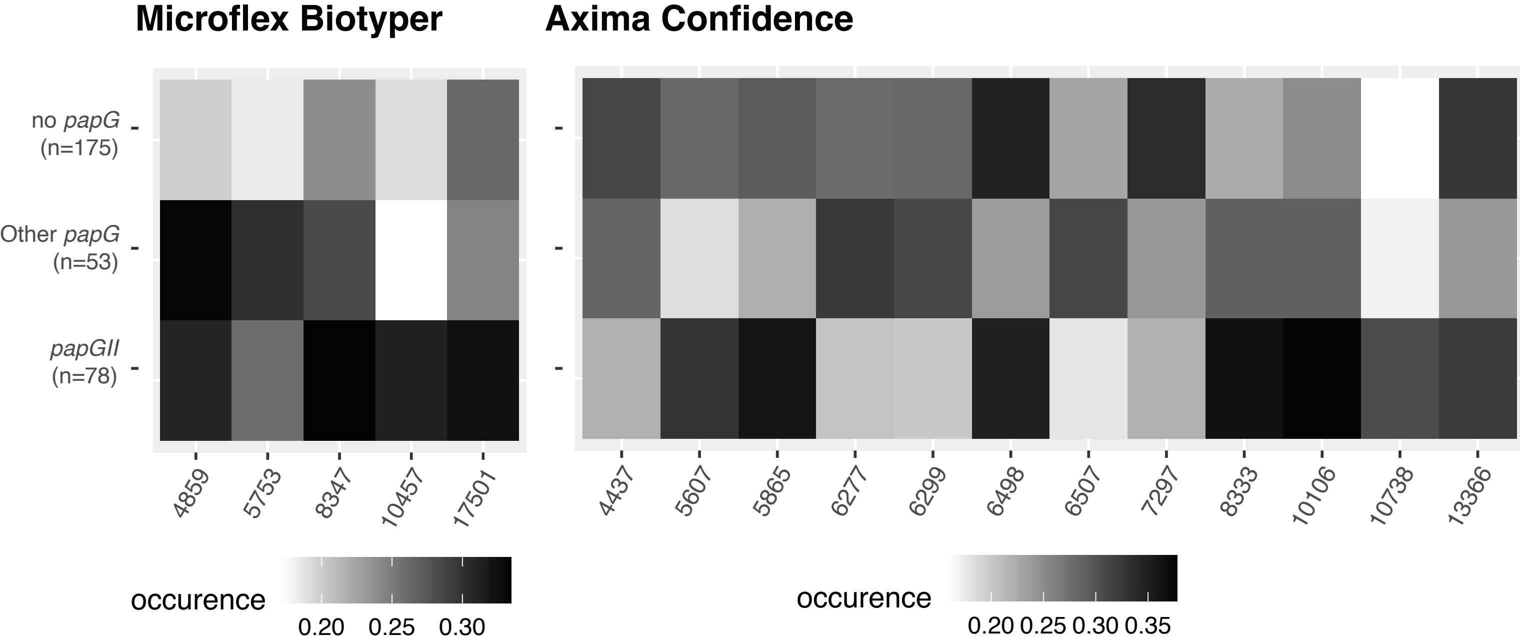

Figure S11: Occurrence of MALDI-TOF mass peaks in spectra acquired from *E. coli* strains encoding no *papG* gene, encoding a *papG* variant other than papGII and encoding *papGII*. 'Occurrence' refers to the percentage of spectra per group in which a peak was detected. Each strain was measured in quadruplicate either on a Microflex Biotyper device, or an Axmina Confidence device. Masses are only depicted if detected in > 30% or < 25% of spectra for one or more of the groups.

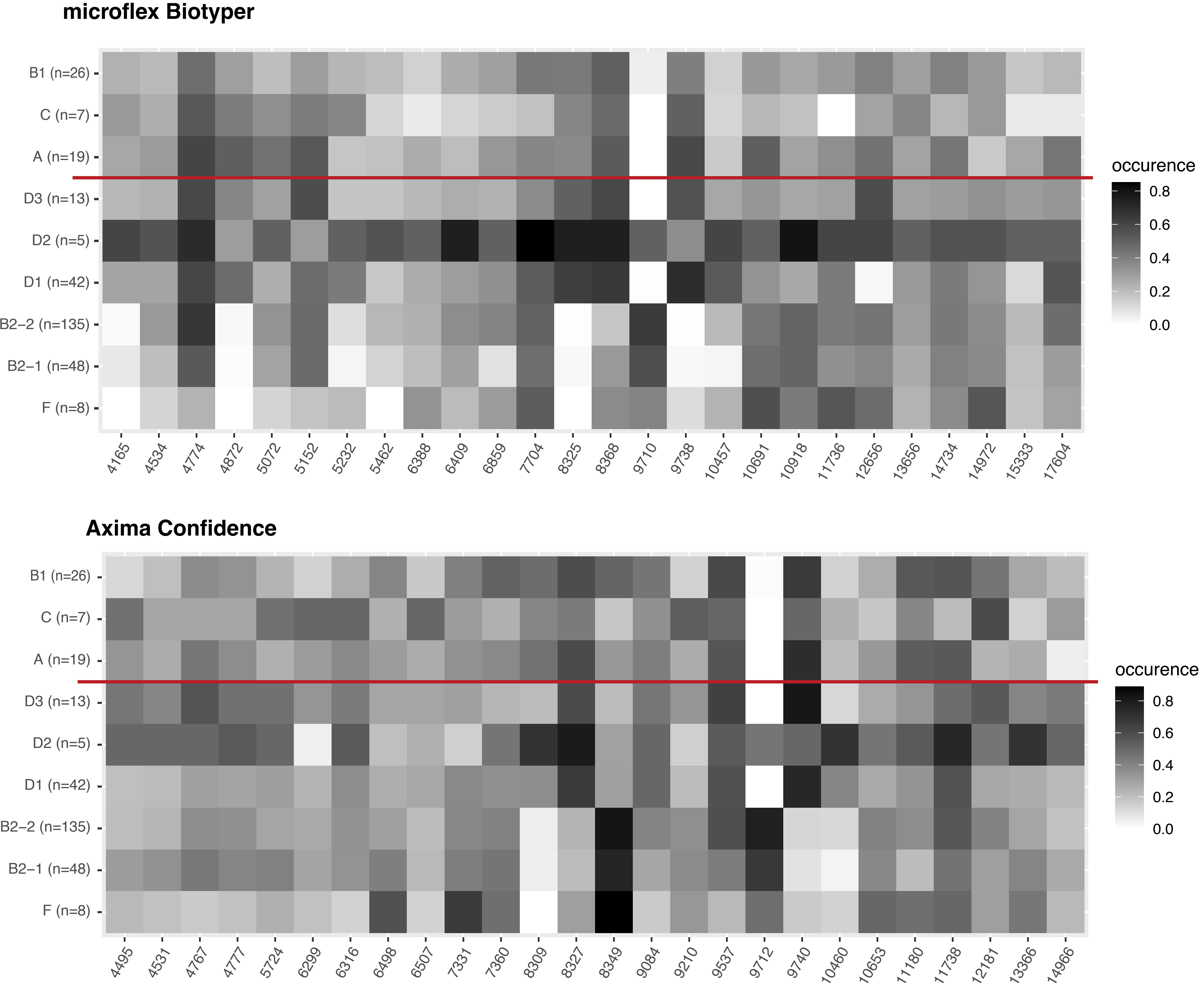

Figure S12: Occurrence of MALDI-TOF mass peaks in spectra acquired from *E. coli* strains of different phylogroups. 'Occurrence' refers to the percentage of spectra per group in which a peak was detected. Each strain was measured in quadruplicate either on a Microflex Biotyper device, or an Axmina Confidence device. Phylogroups for which less than five strains were available (E1, E2 and G) were excluded from the plot. Masses are only depicted if detected in > 50% or < 25% of spectra for one or more of the groups.

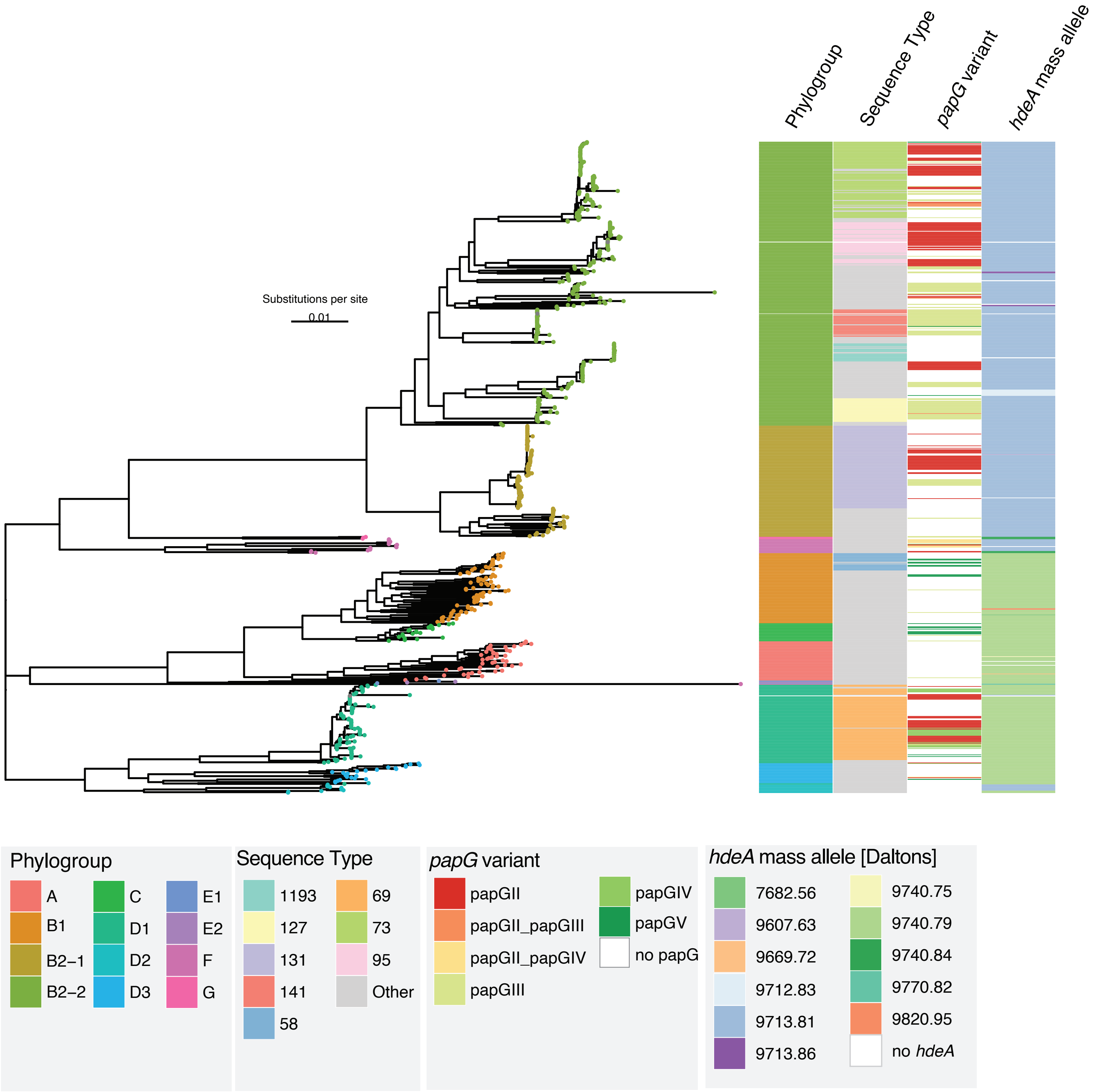

Figure S13: Core genome phylogeny of the *E. coli* strains collected for this study (one strain per clinical case, n=825). Phylogroup assignment, Sequence Type (ST) (eight most frequent ones coloured, more rare STs in grey), *papG* variant, mass of HdeA, predicted from the amino acid sequence.

a

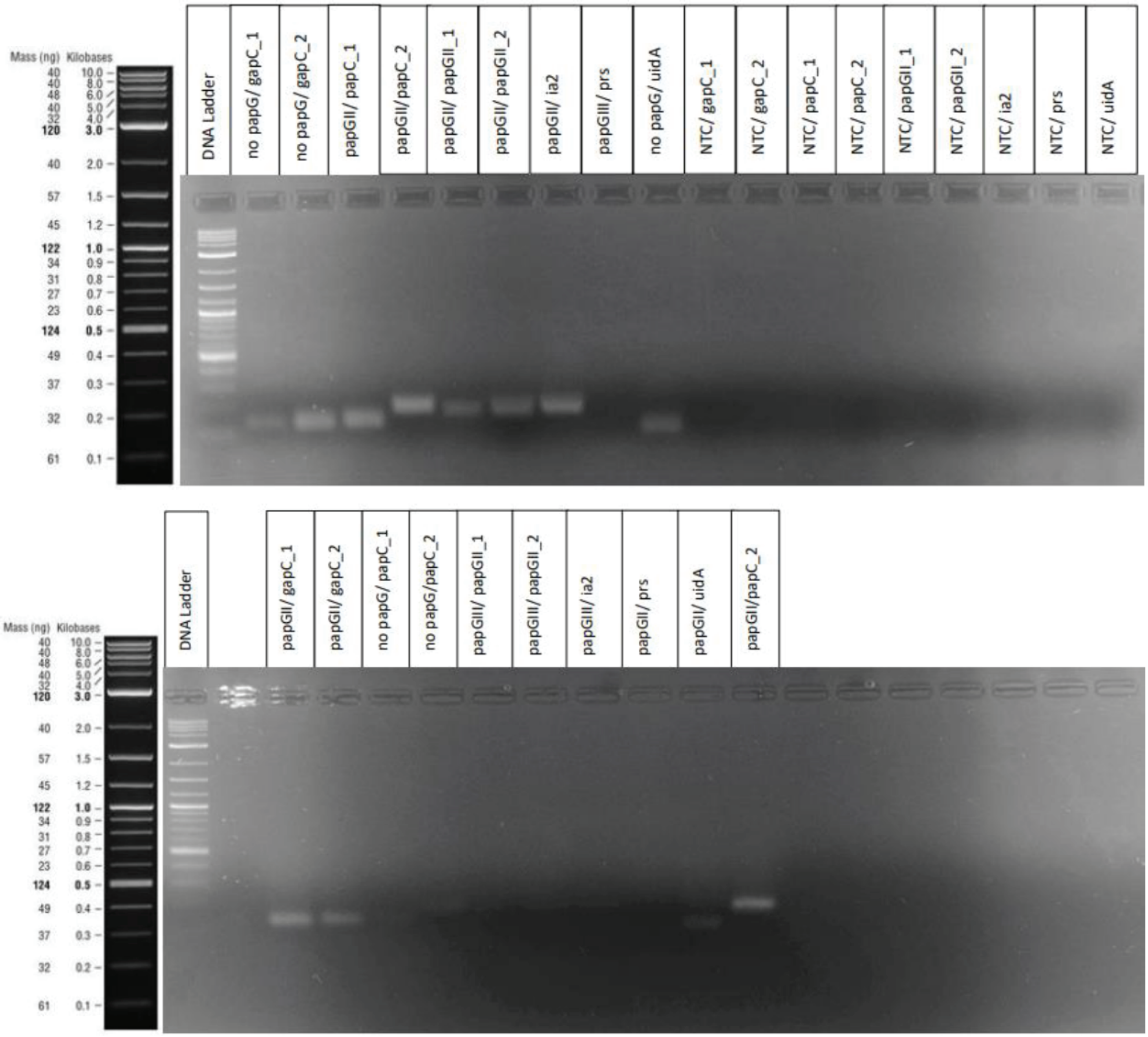

b

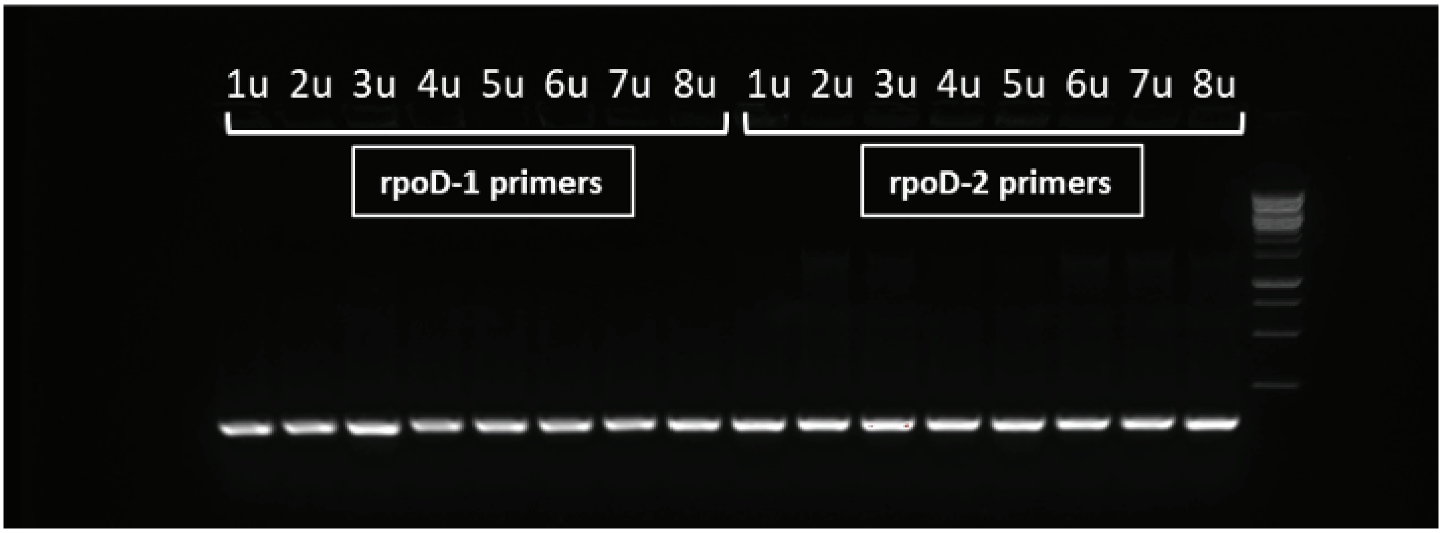

Figure S14: Results of the endpoint PCR assay (a) to test the functionality of the primers designed at centre 1. This also includes tests for the crossreactivity between papGII and papGIII primers. (b) to test the functionality of the *rpoD* primers designed at centre 2.

a

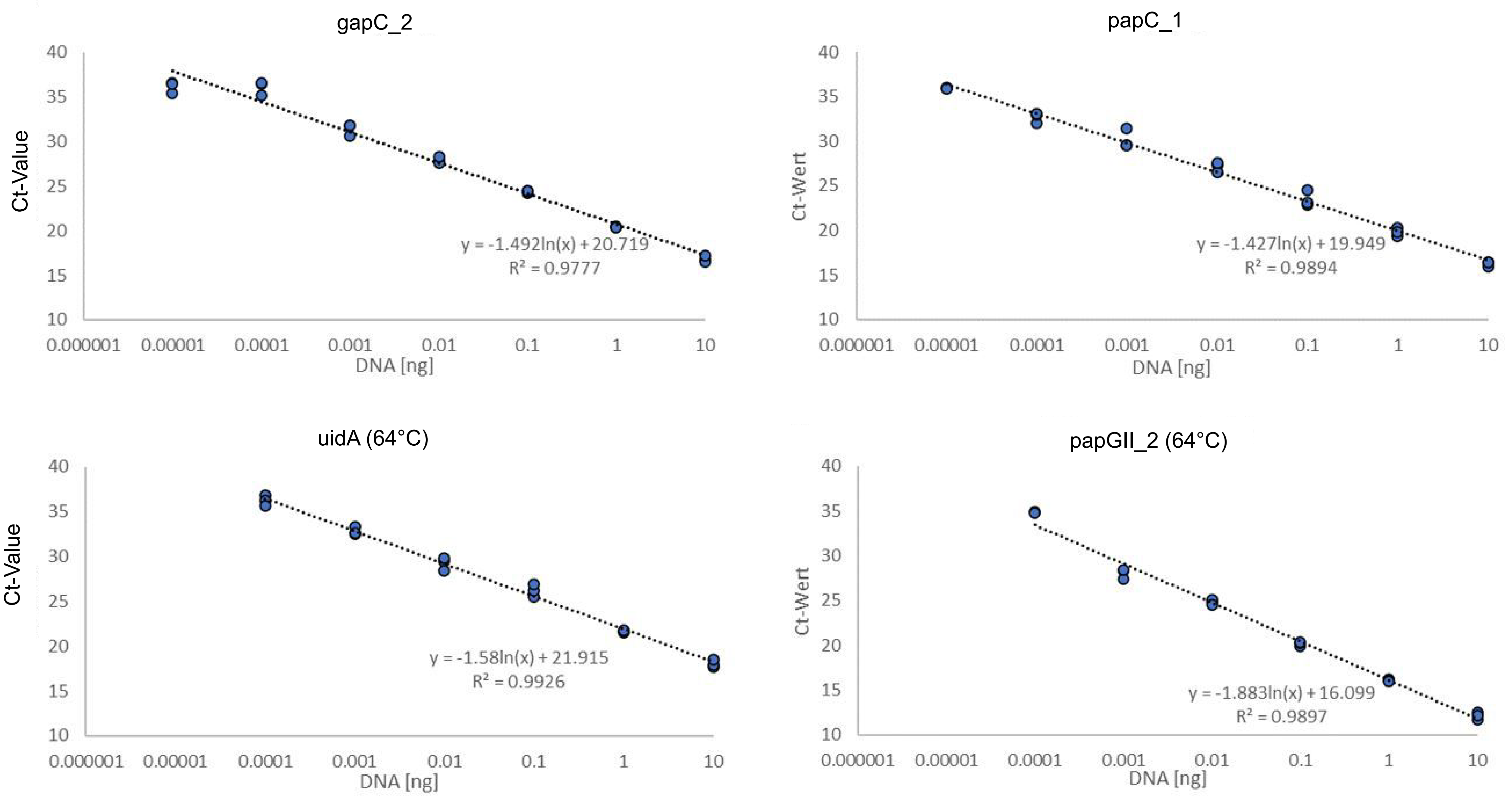

b

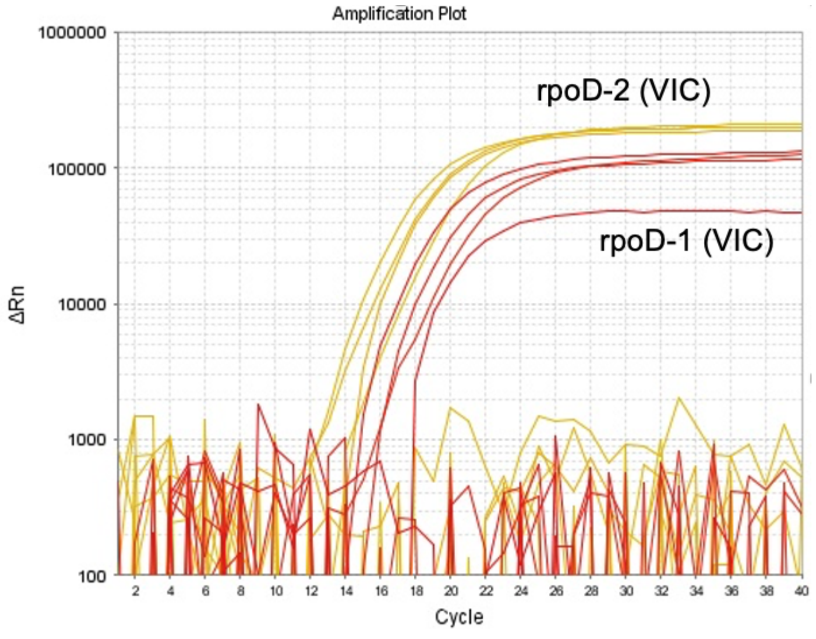

Figure S15: Evaluating the efficiency of primers and probes used in our qPCR assay (**a**) qPCR standard curves and values for the primer pairs gapC_2, papC_1, uidA and papGII_2 tested at centre 1. Each measurement was performed in triplicate. (**b**) Amplification plots for the two rpoD probes designed at centre 2. Measurements performed in quadruplicate.

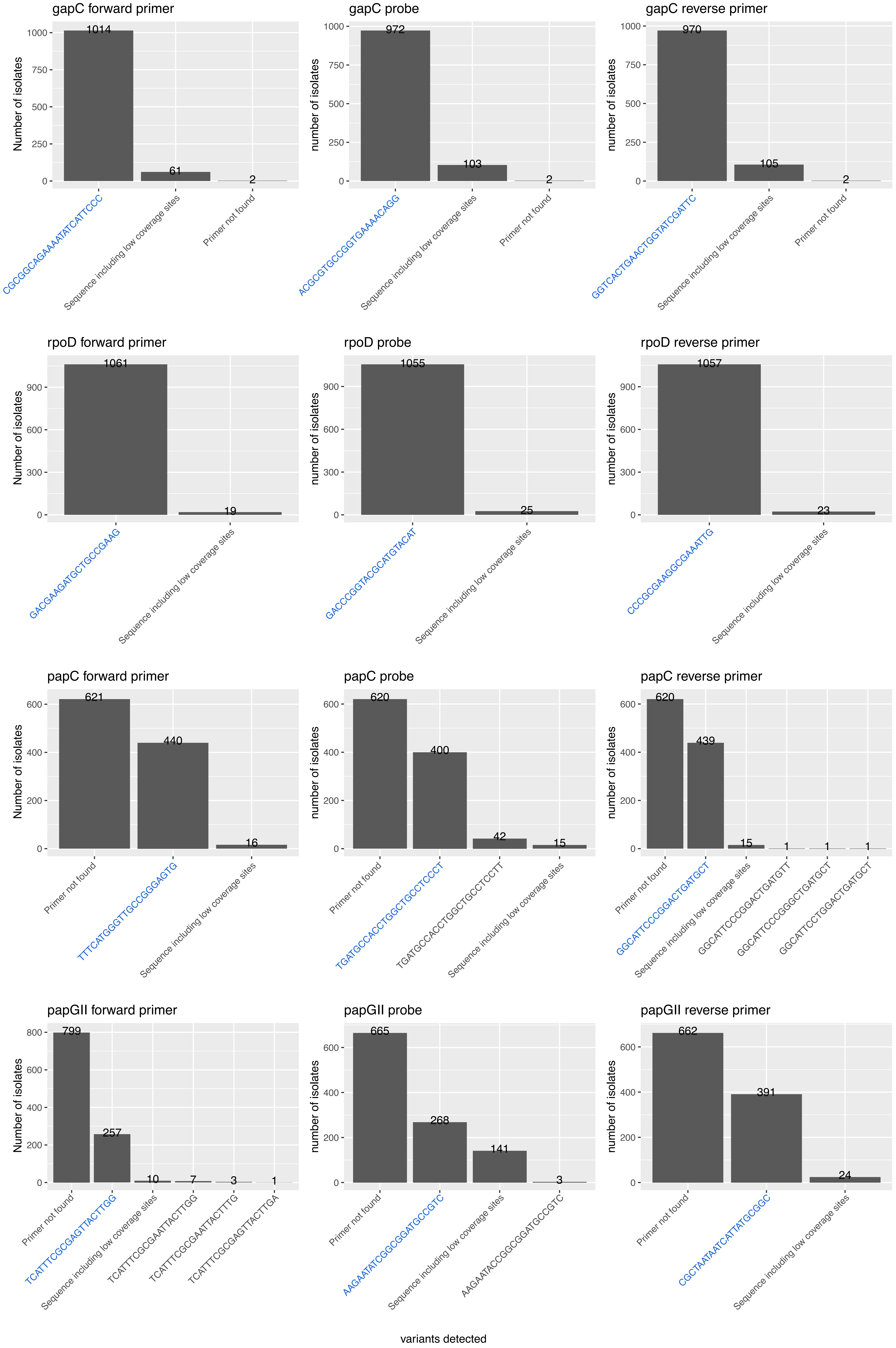

Figure S16: Variants of primer and probe sequences detected in our genome collection (n=1,076). Sequences used in the qPCR assay are indicated in blue and alternative variants detected in the genomes are depicted in black. Variants were called using the variantcaller Freebayes via snippy and using a minimum coverage of 20x.

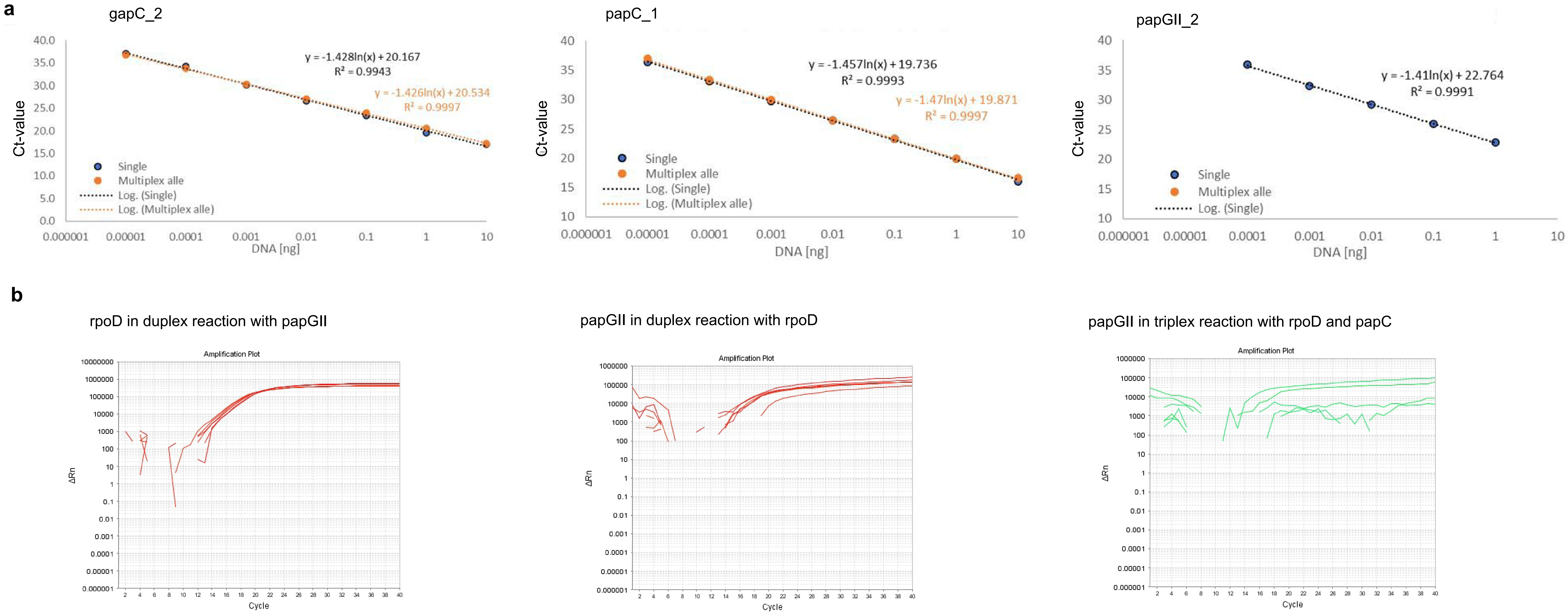

Figure S17: (a) Efficiency of the primer pairs in the single reaction (blue) and in a triplex reaction (orange) for the primers used at center 1 (gapC, papC and papGII). **(b)** Amplification curves of primers used at center 2: rpoD and papGII in duplex reactions and of papGII in a triplex reaction with rpoD and papC.

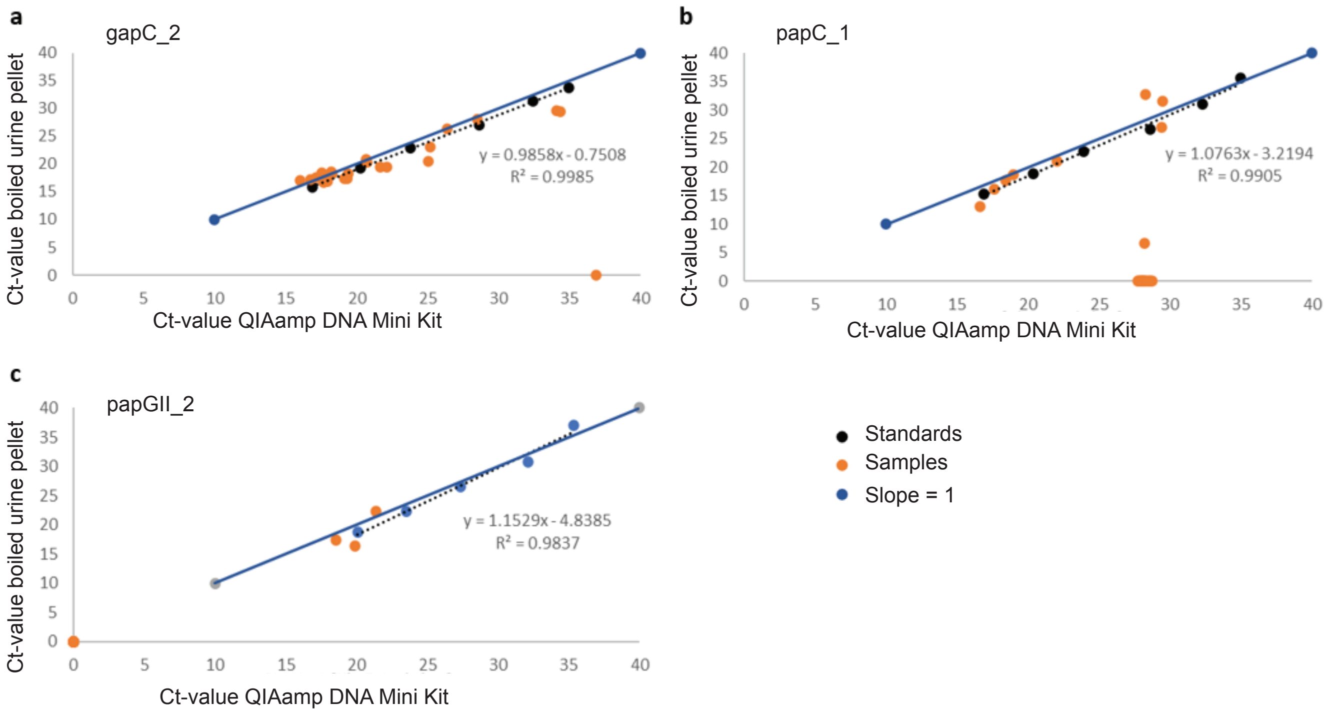

Figure S18: Comparison of the Ct-value yielded when processing urine pellets (n=24) using the QIAamp DNA Mini Kit and after boiling for 10 minutes.

Supplementary Methods:

Endpoint PCR

Centre 1

To test the functionality of the selected primers we performed an endpoint PCR. The primers used are summarised in **Table 1**. Six primer pairs were newly designed for this study using Primer-BLAST (4) and three were retrieved from previous publications (1,2).

Table 1: Primers which were used in the endpoint PCR assay at centre 1. Primers with an asterisk (*) were newly designed for this study.

**Name**	**Target**	**Amplified [bp]**	**Temp. [°C]**	**Sequence (5‘-3‘)**
gapC_1*	*gapDH-C*	140	64	F: TGA TTC AAA CTA TGG TCC GTT CCC<br>R: CAA TGA TTT CTG CAC CTT TCG C
gapC_2*	*gapDH-C*	143	64	F: CGC GGC AGA AAA TAT CAT TCC C<br>R: GAA TCG ATA CCA GTT CAG TGA CC
papC_1*	*papC*	109	64	F: TTT CAT GGG TTG CCG GGA GTG<br>R: AGC ATC AGT CCG GGA ATG CC
papC_2*	*papC*	152	64	F: GGG AGT GGA TAT TCG TCC TGA<br>R: ACT GAA CTG ATG AGA GTC TCC TC
papGII_1*	*papGII*	142	64	F: ATT GTC TTT TAC TCC CTT GG<br>R: CCA CGC AAT ATA CTC CCT
papGII_2*	*papGII*	142	59/64	F: TCA TTT CGC GAG TTA CTT GG<br>R: GCC GCA TAA TGA TTA TTA GCG
Ia2-383f /ia2-572r	*papGII*	190	64	F: GGG ATG AGC GGG CCT TTG AT<br>R: CGG GCC CCC AAG TAA CTC G
prs-198f/ prs-455r	*papGIII*	214	64	F: GGC CTG CAA TGG ATT TAC CTG G<br>R: GGC CTG CAA TGG ATT TAC CTG G
uidA405-F/uidA-R	*uidA*	230	59/64	F: CAA CGA ACT GAA CTG GCA GA<br>R: CAT TAC GCT GCG ATG GAT

Table 2 summarises the composition of the endpoint PCR reactions and **Table 3** the thermocycler program used. The concentration of the genomic DNA was determined using Qubit (Invitrogen, Waltham, USA) and ranged between 21ng/µl - 114ng/µl.

Table 2: Composition of the Endpoint PCR reactions

Component	Volume [µl]	Finale Concentrations
5x Reaction Buffer	5.0	1X
dNTPs [10 mM]	0.5	200 µM
Q5 High-Fidelity DNA-Polymerase	0.25	0.02 U/µL
Forward Primer [10µM]	1.25	0.5 µM
Reverse Primer [10µM]	1.25	0.5 µM
Template DNA	2	variable
Nuclease-free Water	14.75	
Total	25	

Table 3: Thermocycler program used for the Endpoint PCR reaction.

Process	Temperature [°C]	Time [seconds]	Cycles
Initial denaturation	98	30	1
Denaturation	98	10	32
Primer annealing	64/59	15	32
Elongation	72	120	1
Finale extension	72	120	1
Hold	12	∞	1

Using the endpoint PCR design summarised in **Table 4**, we assessed the functionality of the primers and tested for cross-reactions between the primers designed for *papGII* and *papGIII*, as there are the two *papG* variants which are genetically most similar (3).

Table 4: Endpoint PCR experimental design to test for the functionality of all primers and the cross reactivity between the primers for *papGII* and *papGIII*.

Name	Target	*E. coli* strain	*papG* variant	Expected Outcome
gapC_1	*gapDH-C*	113269-19	no papG	+
gapC_2	*gapDH-C*	113269-19	no papG	+

papC_1	*papC*	700223-20	papGII	+
papC_2	*papC*	700223-20	papGII	+
papGII_1	*papGII*	113269-19	papGII	+
papGII_2	*papGII*	113269-19	papGII	+
Ia2-383f /ia2-572r	*papGII*	113269-19	papGII	+
prs-198f/prs-455r	*papGIII*	711763-19	papGIII	+
uidA405-F/uidA-R	*uidA*	113269-19	no papG	+
gapC_1	*gapDH-C*	145484-19	papGII	+
gapC_2	*gapDH-C*	145484-19	papGII	+
papC_1	*papC*	113269-19	no papG	-
papC_2	*papC*	113269-19	no papG	-
papGII_1	*papGII*	711763-19	papGIII	-
papGII_2	*papGII*	711763-19	papGIII	-
Ia2-383f /ia2-572r	*papGII*	711763-19	papGIII	-
prs-198f/prs-455r	*papGIII*	145484-19	papGII	-
uidA405-F/uidA-R	*uidA*	711763-19	papGII	+
papC_1	*papC*	700223-20	papGII	+

The resulting PCR products were separated using a 2% agarose gel electrophoresis in 1X TAE buffer solution, at 40 Volt during 50 minutes. We used the 'Safe Red' dye to visualise the products and recorded the gel on a 'Gel Visualizer Fusion X'.

Centre 2

At centre 2, two sets of primers and probes for the *E. coli* core gene *rpoD* were newly designed using Primer-BLAST (4) (**Table 5**). Both sets were tested for their functionality using endpoint PCR. We used 1 µl of *E. coli* frozen stocks (*E. coli* strains isolated in routine diagnostics and preserved in 1ml of skim milk media) as template.

Table 5: Primers which were used in the endpoint PCR assay at centre 2. Primers with an asterisk (*) were newly designed for this study.

Name	Target	Temp. [°C]	Sequence (5'-3')

rpoD_1*	*rpoD*	59<br><br>59.5	F: GAC GAA GAT GCT GCC GAA G<br>R: CAA TTT CGC CTT CGC GGG<br>Probe: TTC AAC GGT GCC CAT TTC AC
rpoD_2*	*rpoD*	59<br><br>58.5	F: AGA TGC TGC CGA AGC CG<br>R: TAG CGA TGT CAA TTT CGC CTT<br>Probe:ATG TAC ATG CGT ACC GGG TC

Quantitative PCR

Centre 1

In the next step, we evaluated the performance of our primers in a quantitative PCR (qPCR) assay. **Table 6** summarises the composition of the qPCR reactions and **Table 7** summarises the thermocycler program used.

Table 6: Composition of the qPCR assay performed at centre 1

**Component**	**Volume [µl]**	**Finale Concentrations**
Luna Universal Probe qPCR Master Mix*	10	1X
Probe [10µM]	0.4	0.2 µM
Forward Primer [10µM]	0.8	0.4 µM
Reverse Primer [10µM]	0.8	0.4 µM
Template DNA	1	variable
Nuclease-free Water	7	
Total	20	

Table 7: Thermocycler program used for the qPCR reaction performed at centre 1

**Process**	**Temperature [°C]**	**Time [seconds]**	**Cycles**
Initial denaturation	95	60	1
Denaturation	95	15	40
Primer annealing	64/59	15	40
Extension	60	30	40

We tested the specificity of the primers gapC_2, uidA, papC_1 and papGII on 32 strains (**Table 8**).

Table 8: 32 strains used to assess the specificity of the selected PCR primers uidA, gapC_2, papC_1 and papGII_2. 'ST' = Sequence Type.

Strain	Phylogroup	ST	H-ype	O-type	Capsule Type	papG variant
113269-19	A	1972	H30	O147	no capsule	no papG
103713-49	B1	58	H25	O9	no capsule	no papG
100223-19	B2	73	H1	O25	KX21	no papG
128632-18	C	1426	H16	O8	no capsule	no papG
100080-19	D	362	H31	O23	KX75	no papG
712184-19	E	1011	H45	O166	no capsule	no papG
117680-19	F	3058	H24	O8,O112	no capsule	no papG
702833-20	G	1163	H23	O171	no capsule	no papG
145484-19	B2	131	H5	O16	KX05	papGII
700223-20	B2	131	H4	O25	KX21	papGII
106880-19	B2	131	H4	O25	KX41	papGII
107962-20	B2	131	H4	O25	no capsule	papGII
100033-19	B2	95	H7	O1	KX03	papGII
100888-20	B2	73	H1	O6	KX29	papGII
123488-18	B2	73	H1	O6	KX41	papGII

711667-19	B2	73	H1	O6	no capsule	papGII
127688-19	D	69	H18	O15	K96	papGII
109841-19	D	69	H4	O117-Gp8	KX31	papGII
721814-18	F	62	H45	O7	KX03	papGII
135695-19	F	648	H2	O153var1	KX42	papGII
711763-19	A	93	H4	O5	KX21	papGIII
130292-18	B1	75	H8	O112	no capsule	papGIII
118504-18	B2	131	H4	O25	KX21	papGIII
102689-19	B2	12	H5	O4	KX53	papGIII
700699-20	C	88	H9	O8	no capsule	papGIII
116138-19	G	117	H4	O143	no capsule	papGIII
103840-19	D	69	H1	O15	KX47	papGIV
701188-20	D	38	H30	O153var1	KX21	papGIV
716875-19	F	59	H7	O1	KX42	papGIV
720707-18	B2	73	H1	O6	KX24	papGV
102916-19	C	367	H19	O9	no capsule	papGV
720603-19	D	69	H18	O15	KX02	papGV

Centre 2

We compared the efficiency of the two newly designed *rpoD* probes in a qPCR assay, using the composition summarised in **Table 9** and the thermocycler program summarised in **Table 10.**

Table 9: Composition of the qPCR assay performed at centre 2

Component	Volume [µl]	Finale Concentrations
Luna Universal Probe qPCR Master Mix*	10	0.48X
Probe [2.5µM]	1	0.12 µM
Forward Primer [20µM]	0.5	0.48 µM
Reverse Primer [20µM]	0.5	0.48 µM
Template (*E. coli* frozen stocks)	1	variable
Nuclease-free Water	8	
Total	21	

Table 10: Thermocycler program used for the qPCR reaction performed at centre 2

Process	Temperature [°C]	Time [seconds]	Cycles
Initial denaturation	95	300	1
Denaturation	95	15	40
Primer annealing	60	10	40
Extension	60	30	40

Multiplexing the qPCR

Centre 1

To further decrease the turnaround time of our assay, we further assessed the possibility to combine our primer to a multiplex assay (composition summarised in **Table 11**).

Table 11: Composition of the qPCR assay, adapted for multiplexing used at centre 1

Component	Volume [µl]	Finale Concentrations

Luna Universal Probe qPCR Master Mix*	10	1X
Forward Primer 1 [10µM]	0.8	0.4 µM
Reverse Primer 1 [10µM]	0.8	0.4 µM
Forward Primer 2 [10µM]	0.8	0.4 µM
Reverse Primer 2 [10µM]	0.8	0.4 µM
Probe 1 [10µM]	0.4	0.2 µM
Probe 2 [10µM]	0.4	0.2 µM
Template DNA	1	variable
Nuclease-free Water	5	
Total	20	

Centre 2

At centre 2, the multiplex assays were performed as depicted in **Table 12**.

Table 12: Composition of the qPCR assay, adapted for multiplexing used at centre 2

**Component**	**Volume [µl]**	**Finale Concentrations**
Luna Universal Probe qPCR Master Mix*	10	0.48X
Probe 1 [2.5µM]	1	0.12 µM
Forward Primer 1 [20µM]	0.5	0.48 µM
Reverse Primer 1 [20µM]	0.5	0.48 µM
Probe 2 [2.5µM]	1	0.12 µM
Forward Primer 2 [20µM]	0.5	0.48 µM
Reverse Primer 2 [20µM]	0.5	0.48 µM
Template (*E. coli* frozen stocks)	1	variable
Nuclease-free Water	6	
Total	21	

Applying qPCR assay directly to urine samples

Centre 1

Urine samples were stored at 4°C and were pelleted as previously described (52). Urine samples (1 ml) were centrifuged (10 minutes, 5,000 rounds per minute (rpm)), the supernatants were removed and the pellets were resuspended in 1ml nuclease free water. Samples were centrifuged again (5 minutes, 10,000 rpm), the supernatants were removed and the resulting pellets were resuspended in 50 µl nuclease free water. Samples were then heated to 99°C on a Thermomixer heat block (Eppendorf, Hamburg, Germany) for 10 min. We assessed the efficiency and the limit of detection our qPCR assay performed on samples processed using this method, and compared them to values resulting from qPCR on extracted genomic DNA.

Screening of patient samples

Centre 1

We prospectively collected 543 urine samples, which we processed to urine pellets before boiling (see methods section). All samples were screened for *gapC* and *papC* in a duplex qPCR approach (see composition in **Table 11** and cycling parameters in **Table 7**). In a second experiment, we screened samples which were culture positive for *E. coli* or for which *gapC* had been detected in the multiplex qPCR for the presence of *papGII* using a single qPCR (see composition in **Table 6** and cycling parameters in **Table 7**). 150 multiplex and 74 single *papGII* reactions were performed in duplicate, the remaining ones as single measurements. Samples for which the duplicate measurements had a difference of > 1.5 Ct values in any of the three targets were excluded from further analysis. For the single PCR of *papGII* we further set a cut-off to 36 cycles. Only samples for which a signal was recorded in less than 36 cycles were considered *papGII* positive. All *papGII* positive measurements were furthermore manually curated and measurements for which the amplifications curve was discontinued were set to negative.

Centre 2

We prospectively collected 1,106 *E. coli* strains, for which an AMR profile was measured in routine diagnostics. Strains were either isolated from urine (n=1,011) or from blood culture (n=95) samples of 886 patients. We used 10µl plastic inoculation needles to transfer bacterial material from Müller-Hinton agar plates to 1ml of skim milk freezing media and stored all strains at -80°C. Frozen stocks were thawed at room temperature before being subjected to qPCR. All samples were initially screened for *rpoD* and *papC* (primers and probes tested at

centre 1). Samples which tested positive for *papC* were further subjected to a duplex qPCR amplifying *rpoD* and *papGII* (see composition in **Table 12** and cycling parameters in **Table 10**). All samples and targets were manually reviewed, using a Ct-value of 25 as threshold and setting samples with discontinued amplification curves to negative. Measurements which were tested negative for *rpoD* were excluded from further analyses.

Supplementary PCR Data:

Evaluation of primer functionality

We tested the functionality of eleven primer pairs using an endpoint PCR. For all primer pairs except for prs-198f/prs-455r the expected signal could be recorded and no cross reactivity between the *papGII* primers and *papGIII* target was detected (**Figure S14**). prs-198f/prs-455r was excluded from further analysis. In a next step, we evaluated the limit of detection and efficiency of the remaining primers in a qPCR assay (**Table S3**, **Figure S15**) and tested the specificity of the primers gapC_2, uidA, papC_1, and papGII_2 using 32 E. coli strains of which 24 carried a pap operon and 12 the papG variant papGII. An amplification was recorded in all expected reactions (100/100) and no unexpected signal was recorded using the papC primers (8/8) nor the papGII_2 primers (20/20). Based on the recorded values we selected the primers gapC_2, papC_1, and papGII_2 for further analysis at centre 1 and rpoD_2, papC_1, and papGII_2 at centre 2 (**Table S4**).

Table S4: Primer pairs designed for and included in this study. 'For.'= Forward primer; 'Rev.'= Reverse primer.

Primer	Nucleotide sequence	Annealing temperature	Used at
gapC_2	For.: CGC GGC AGA AAA TAT CAT TCC C<br>Rev.: GAA TCG ATA CCA GTT CAG TGA CC<br>Probe: ACG CGT GCC GGT GAA AAC AGG	64°C	Centre 1
rpoD_1	For.: GAC GAA GAT GCT GCC GAA G<br>Rev.: CAA TTT CGC CTT CGC GGG<br>Probe: ATG TAC ATG CGT ACC GGG TC	60°C	Centre 2
papC_1	For.: TTT CAT GGG TTG CCG GGA GTG<br>Rev.: AGC ATC AGT CCG GGA ATG CC<br>Probe:TGA TGC CAC CTG GCT GCC TCC CT	64°C	Centre 1 & 2
papGII_2	For.: TCA TTT CGC GAG TTA CTT GG<br>Rev.: GCC GCA TAA TGA TTA TTA GCG<br>Probe: AAG AAT ATC GGC GGA TGC CGT C	64°C	Centre 1 & 2

We examined whether variants of these sequences occur in our set of genomes (n=1,076). In >90% of sequences in which all sites of the target had a coverage exceeding the threshold from the variant caller, an exact match of the primer and probe sequences was detected (**Figure S16**). We further assessed the possibility of multiplexing our primers: *gapC*, *rpoD* and *papC* were reliably amplified in duplex reactions (gapC_2/papC_1, rpoD_1/papGII_2) as well as in triplex reactions (gapC_2/papC_1/papGII_2 or rpoD/papC_1/papGII_2) (**Figure S17**). In contrast, whole papGII was reliably amplified in duplex reactions (rpoD_1/papGII_2), it was not reliably amplified in a triplex reaction (neither gapC_2/papC_1/papGII_2 nor rpoD/papC_1/papGII_2) (**Figure S17**). We therefore used gapC_2 and papC_1 in a duplex reaction and screened for *papGII* either in a single reaction (centre 1) or in a duplex reaction amplifying *rpoD* (centre 2). We aimed to apply our assay directly to pelleted urine samples, thereby omitting overnight culturing. We observe a 10-fold increase in the limit of detection for papC_1 and papGII_2 (**Table S3**) doing so compared to when using extracted DNA. When comparing pelleted urine samples (n=24) which were boiled to those whose DNA was extracted using the QIAamp DNA Mini Kit, we observe a high correlation between the resulting Ct-values (**Figure S18**). Only for the papC_1 primer, however, a signal was detected for extracted DNA but not for boiled urine pellets in 11/24 samples, indicating a reduced sensitivity for the papC_1 primers. The main goal of this analysis was the detection of *papGII*, which does not appear to be affected by differences in sample preparation. Considering this and the shorter hands-on time, we decided to boil urine sample pellets at Center 1 and perform the PCR directly from colonies at Center 2 when screening for *gapC*, *papC* and *papGII.* It is therefore possible that the reduced sensitivity for *papC* might be reflected in our screening results, which is certainly a limitation of this study.
